# High-throughput sequence analysis reveals variation in the relative abundance of components of the bacterial and fungal microbiota in the rhizosphere of *Ginkgo biloba*

**DOI:** 10.7717/peerj.8051

**Published:** 2019-11-15

**Authors:** Rujue Ruan, Zhifang Jiang, Yuhuan Wu, Maojun Xu, Jun Ni

**Affiliations:** Hangzhou Normal University, Key Laboratory of Hangzhou City for Quality and Safety of Agricultural Products, College of Life and Environmental Sciences, Hangzhou, China; Hangzhou Normal University, Zhejiang Provincial Key Laboratory for Genetic Improvement and Quality Control of Medicinal Plants, Hangzhou, China

**Keywords:** Fungi, Bacteria, Genus, Ginkgo, Rhizosphere, Soil

## Abstract

**Background:**

The narrow region of soil, in contact with and directly influenced by plant roots, is called the rhizosphere. Microbes living in the rhizosphere are considered to be important factors for the normal growth and development of plants. In this research, the structural and functional diversities of microbiota between the *Ginkgo biloba* root rhizosphere and the corresponding bulk soil were investigated.

**Methods:**

Three independent replicate sites were selected, and triplicate soil samples were collected from the rhizosphere and the bulk soil at each sampling site. The communities of bacteria and fungi were investigated using high-throughput sequencing of the 16S rRNA gene and the internal transcribed spacer (ITS) of the rRNA gene, respectively.

**Results:**

A number of bacterial genera showed significantly different abundance in the rhizosphere compared to the bulk soil, including *Bradyrhizobium*, *Rhizobium*, * Sphingomonas*, *Streptomyces* and *Nitrospira*. Functional enrichment analysis of bacterial microbiota revealed consistently increased abundance of ATP-binding cassette (ABC) transporters and decreased abundance of two-component systems in the rhizosphere community, compared to the bulk soil community. In contrast, the situation was more complex and inconsistent for fungi, indicating the independency of the rhizosphere fungal community on the local microenvironment.

## Introduction

Microorganisms act not only as pathogens, causing plant diseases (Chisholm et al., 2006), but also as neighbors or symbionts, playing important roles in plant productivity and health by providing a plethora of functional activities. Plant-associated microorganisms are often referred to as the plant’s second genome and have received substantial attention in recent years (Berg et al., 2014; Turner, James & Poole, 2013). For studying the microbial diversity of prokaryotes, such as bacteria, PCR amplification of the ubiquitous 16S ribosomal RNA (rRNA) gene, coupled with high-throughput sequencing technologies, have allowed identification of even rare non-culturable microbial species in a sample (Caporaso et al., 2011; Youssef et al., 2009). For studying eukaryotic microbes, such as fungi, the hypervariable internal transcribed spacer (ITS) rDNA sequences are often used to assess taxonomic diversity (Bachy et al., 2013; Bengtsson-Palme et al., 2013).

The narrow region of soil, in contact with and directly influenced by plant roots, is called the rhizosphere. The rhizosphere is critical to normal plant growth and development, with all the inorganic and organic substances exchanged between the root and soil occurring through this zone. On the other hand, plants also modify the rhizosphere to better adapt to the ever-changing environment (Ryan, Delhaize & Jones, 2001). Significant differences have been reported with respect to the physical, chemical and biological characteristics of the plant rhizosphere soil compared to the surrounding bulk soil, and this phenomenon is known as the “rhizosphere effect” (Hartmann, Rothballer & Schmid, 2008). Specifically, enormous numbers of microbes live in the rhizosphere, forming a complex plant-associated microbial community, and this community is considered to be crucial for plant health (Berendsen, Pieterse & Bakker, 2012).

*Ginkgo biloba*, a “living fossil”, is an important long-lived native Chinese tree species with no living relatives (Zhou, 2009). It is used as a medical plant, with ginkgo herb being commonly used as an herbal dietary supplement and for the treatment of many ailments, including Alzheimer’s disease (Rimbach et al., 2001). It is also one of the horticultural tree species which is now widely planted in China. Moreover, *G. biloba* is regarded as a valuable municipal tree in many cities in China (Fig. S1, Table S1). Due to its importance to human health and the environment, much research has been carried out on ginkgo, especially focused on the flavonoid biosynthesis pathway in ginkgo leaves (Ni et al., 2018a; Ni et al., 2018b; Ni et al., 2017). However, our knowledge of the relationship between ginkgo and the microbiota in its rhizosphere is still limited.

In this research, the 16S and ITS sequences were analyzed in bacteria and fungi, respectively, to estimate the diversity of microbiota in the ginkgo root rhizosphere compared to the bulk soil. A number of bacterial genera were found to accumulate in the rhizosphere, while there were also some bacterial genera for which the abundance decreased in rhizosphere. In contrast, the situation with the abundance of fungal genera in the rhizosphere versus the bulk soil was complex and inconsistent across the three sampling sites, indicating that fungal abundance was relatively independent of the rhizosphere microenvironment. Our results identified a number of bacterial genera, the abundance of which differed between the rhizosphere and the bulk soil, indicating a complex relationship between ginkgo and soil microbes.

## Materials & Methods

### Sampling and DNA extraction

Three independent replicate sites in the campus of Hangzhou Normal University were selected for soil sample collection in August 2017. Ginkgo roots were collected at a depth of about 20 cm below ground level. Large soil aggregates were removed by shaking the roots, and the rhizosphere soil was defined as the remaining soil particles adhering to the roots. The bulk soil was collected about 10 m away from the ginkgo trees at a depth of about 20 cm below ground level. The bulk soil was also free from roots of other plants. For the collection of rhizosphere soil, roots were transferred to a 15 ml-Falcon tube containing 2.5 ml phosphate-buffered saline (PBS) solution, and were sonicated 30 times (Scientz-JY92-IIN; Scientz, Ningbo, China), each consisting of 30 s pulses at 160 W, with breaks between pulses of 30 s. After removing the roots, the suspension was centrifuged at 1,500 × g for 20 min, and the pelleted soil was used for DNA extraction. The PowerSoil™ DNA Isolation Kit (MoBio) was used to extract the DNA from the rhizosphere and bulk soil samples, following the manufacturer’s instructions.

### PCR amplification and sequencing of amplicon libraries

The DNA samples were individually amplified by PCR using primers S-D-Bact-0341-b-S-17 (5′-CCTACGGGNGGCWGCAG-3′), S-D-Bact-0785-a-A-21 (5′-GACTACHVGGGTATCTAATCC-3′) for 16S rDNA in bacteria (Klindworth et al., 2012), and ITS1 (5′-TCCGTAGGTGAACCTGCGG-3′), ITS2 (5′-GCTGCGTTCTTCATCGATGC-3′) for ITS in fungi (Sreenivasaprasad et al., 1996). Each 30 µl PCR reaction mixture contained 5∼10 ng DNA template, 15 µl 2 × Master Mix (Phusion® High-Fidelity PCR Master Mix with GC Buffer, New England Biolabs, USA), with each primer in the reaction mixture being supplied at a concentration of 3 µM. Cycling conditions included initial denaturation at 98 °C for 1 min, followed by 30 cycles of denaturation at 98 °C, each cycle lasting 10 s, annealing at 50 °C for 30 s, and extension at 72 ° C for 30 s; a final extension phase was performed at 72 °C for 5 min. The PCR products were separated on a 2% (w/v) agarose gel. The DNA bands between 400 bp and 450 bp were collected. The DNA was extracted from the gel slices using the GeneJET gel extraction kit (Thermo Scientific, USA). The amplicon libraries were constructed using NEB Next® Ultra™ DNA Library Prep Kit for Illumina (New England Biolabs) according to the manufacturer’s protocol. The qualified libraries were sequenced on an Illumina Hiseq2500 platform and 250 bp paired-end reads were generated. The raw sequencing data have been deposited in NCBI Sequence Read Archive (SRA) under accession number PRJNA565829 for bacteria, and PRJNA566252 for fungi.

### Sequencing data analysis, OTU production and annotation

The original paired-end reads, cutting off the barcode and primer sequences, were merged to total tags with FLASH (v.1.2.11) (Magoc & Salzberg, 2011). The total tags were filtered by Qiime (v.1.9.1) (Bokulich et al., 2013; Caporaso et al., 2010). These tags were then compared with the reference database (Gold database, http://drive5.com/uchime/uchime_download.html) using UCHIME algorithms (http://www.drive5.com/usearch/manual/uchime_algo.html) to detect and remove the chimera sequences (Edgar et al., 2011; Haas et al., 2011). After that, these tags were termed effective tags and were ready for further analysis.

The effective tags with ≥97% similarity were assigned to the same operational taxonomic units (OTUs) using Uparse (v8.1.1861), and the sequence with the highest frequency of occurrence in each OTU was selected as the representative sequence for further annotation (Edgar, 2013). For each representative sequence, the annotation was performed using the uclust method and the Silva database to the level of kingdom, phylum, class, order, family, genus and species, to determine the community composition of each sample. OTU abundance information was normalized using a standard of sequence number corresponding to the sample with the least sequences (For bacteria, the number was 22,755. For fungi, the number was 34,819). Subsequent analyses were all performed based on these normalized data.

### Alpha and beta diversity analysis

Alpha diversity was applied to analyze complexity of species diversity for a community through four indices, including abundance-based coverage (ACE) index, Shannon diversity index, phylogenetic diversity (PD)_whole_tree and Good’s coverage index. All these indices from our samples were calculated with QIIME (Version 1.9.1) and displayed using R software (Version 3.2.2). The ACE estimator (http://www.mothur.org/wiki/Ace) was selected to determine community richness, while the Shannon index (http://www.mothur.org/wiki/Shannon) was used to estimate community diversity, and Good’s coverage index (http://www.mothur.org/wiki/Coverage) was used to quantify sequencing depth.

For beta diversity analysis, principal component analysis (PCA) was applied to reduce the dimension of the original variables using the FactoMineR package and ggplot2 package in R software (Version 3.2.2; R Core Team, 2015). Unweighted Pair-Group Method with Arithmetic Means (UPGMA) Clustering was performed as a type of hierarchical clustering method to interpret the distance matrix using average linkage, and was conducted using QIIME software (Version 1.9.1).

### Correlation analysis and KEGG functional analysis

Correlation analysis (using Spearman’s rank correlation analysis) was performed based on the changes in species abundances in different communities, using CCREPE (http://huttenhower.sph.harvard.edu/ccrepe). The first 100 groups were displayed by Cytoscape (http://chianti.ucsd.edu/cytoscape-3.2.1/). To analyze the function of the microbiota, Tax4Fun (http://tax4fun.gobics.de) was used to determine the Kyoto Encyclopedia of Genes and Genomes (KEGG) estimate. The variation analysis was carried out based on the functional abundance of the samples.

## Results

### Quality metrics of microbiome sequencing data from the rhizosphere and bulk soil samples

To investigate the differences in microbiota between the rhizosphere of ginkgo roots and the bulk soil, 16S rDNA amplicon sequencing for bacteria and ITS rDNA sequencing for fungi were performed on DNA samples extracted from the ginkgo rhizosphere and from bulk soil collected 10 m from the nearest ginkgo tree. Three independent replicate sites were chosen for sample collections. Within each site, triplicate samples were collected from both the rhizosphere and the bulk soil. For bacterial 16S rDNA analysis, more than 40,000 total tags were obtained in each sample except for S-2 (38,717 total tags) in site 1, and R-4 (34,905 total tags) in site 2. After trimming and filtering, more than 80% of the tags were assigned to be effective tags that were ready for further analysis (Table 1). For fungal ITS rDNA analysis, more than 40,000 total tags were obtained in each sample except for S-8 (36,779 total tags) in site 3. Similarly, more than 85% of the tags were assigned to be effective tags that were ready for further analysis. In addition, more than 98% base calls were 99% confidence (Q20) and more than 94% base calls were 99.9% confidence (Q30) for each sample with respect to both bacteria and fungi. These results indicated that the sequencing data from 16S and ITS were acceptable (Table 1).

**Table 1:** The raw sequencing data and quality control of 16S and ITS. R-1 to R-9 are samples collected from the rhizosphere. S-1 to S-9 are samples collected from the bulk soil. AvgLen, average lengths of the clean reads. Q20 and Q30 are the ratio of bases with quality value more than 20 and 30 (error rates of less than 1% and 0.1%, respectively) in the clean reads.

**18 samples from 3 sites**		**16S**						**ITS**					
		**Total reads**	**Clean reads**	**Proportion (%)**	**AvgLen**	**Q20**	**Q30**	**Total reads**	**Clean reads**	**Proportion (%)**	**AvgLen**	**Q20**	**Q30**
**Site 1**	R-1	44,196	38,089	86.18	413	98.63	95.17	44,032	43,394	98.55	226	99.83	99.37
	R-2	41,143	34.371	83.54	413	98.66	95.28	40,872	38,929	95.24	225	99.82	99.30
	R-3	43,319	37,691	87.01	413	98.62	95.10	40,753	39,207	96.21	232	99.81	99.28
	S-1	44,224	39,050	88.30	419	98.52	94.76	43,791	42,005	95.92	249	99.54	98.44
	S-2	38,717	34,300	88.59	419	98.53	94.74	44,216	40,097	90.68	254	99.55	98.49
	S-3	41,748	37,364	89.50	419	98.54	94.82	40,967	35,942	87.73	262	99.55	98.45
**Site 2**	R-4	34,905	30,760	88.12	417	98.45	94.54	44,226	39,959	90.35	252	99.75	99.10
	R-5	41,833	36,680	87.68	416	98.55	94.93	42,358	40,274	95.08	246	99.72	99.00
	R-6	43,612	38,426	88.11	414	98.47	94.70	43,686	42,656	97.64	253	99.68	98.86
	S-4	44,197	39,884	90.24	417	98.50	94.65	42,357	38,430	90.72	266	99.54	98.43
	S-5	42,674	37,796	88.57	418	98.51	94.67	41,487	36,392	87.72	265	99.54	98.44
	S-6	41,380	36,649	88.57	418	98.53	94.79	44,379	41,628	93.80	252	99.67	98.84
**Site 3**	R-7	40,526	33,739	83.25	415	98.58	95.04	43,107	42,305	98.14	234	99.78	99.19
	R-8	40,593	34,748	85.60	415	98.55	94.89	41,706	39,722	95.24	247	99.76	99.13
	R-9	41,556	34,434	82.86	415	98.46	94.62	40,768	37,212	91.28	238	99.83	99.35
	S-7	42,328	37,973	89.71	419	98.54	94.80	41,338	41,156	99.56	258	99.65	98.80
	S-8	42,939	38,061	88.64	418	98.47	94.58	36,779	35,699	97.06	261	99.48	98.30
	S-9	43,066	38,837	90.18	417	98.48	94.66	44,097	42,364	96.07	261	99.67	98.86

### OTU analysis and annotation of microbiome sequences

**Figure 1:**
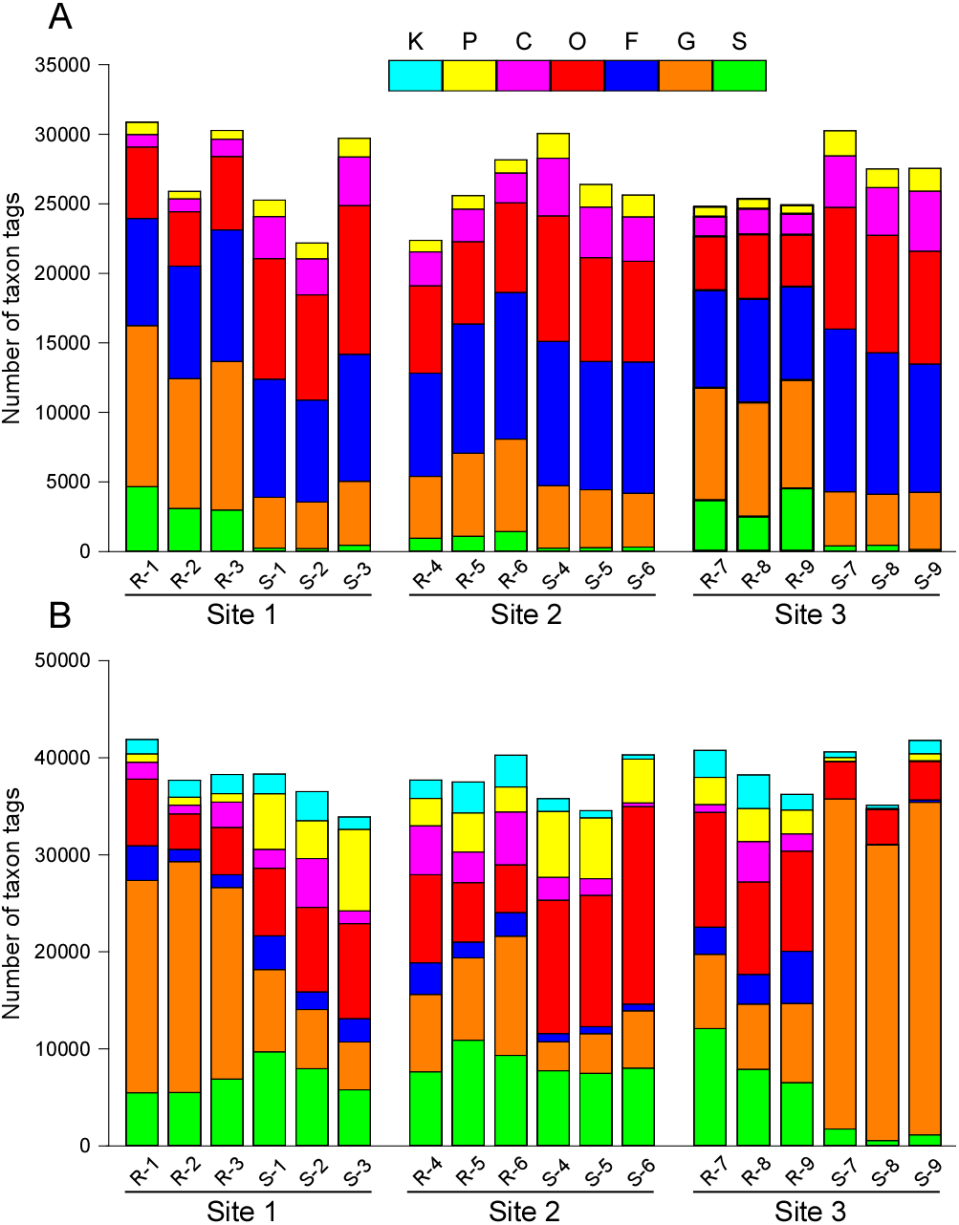
Different levels of taxonomic distribution of taxon tags. Site 1, Site 2 and Site 3 are the three sample collection sites. R-1 to R-9 are samples collected from rhizosphere. S-1 to S-9 are samples collected from the bulk soil. Different colors represent different levels of taxonomy. (A) Bacteria. (B) Fungi. K, kingdom. P, phylum. C, class. O, order. F, family. G, genus. S, species.

To study the species diversities of the microbiome from the sequencing data, the annotated clean reads (taxon reads) were clustered into OTUs. An average (± standard deviation) number of 2823.0 ± 283.7 OTUs was obtained for bacteria, with a corresponding number of 430.8 ± 146.2 OTUs for fungi (Table 2). These taxon tags were also classified to different levels of taxonomy, and three technical replicates from the same soil and site exhibited similar taxonomic patterns, which showed that the repeatability of the experiment was acceptable. Different taxonomic patterns were observed between samples from rhizosphere and bulk soil from each of the three collection sites. This indicated marked differences in the microbiota between the rhizosphere and the bulk soil (Fig. 1, Tables S2 and S3). We also investigated the ten most-abundant items with respect to different taxonomic levels of phylum, class, order, family and genus. For bacteria, the extent of variation increased from phylum to genus (Figs. S2–S6). Specifically, the frequencies of the ten most abundant genera in rhizosphere soil were quite different in the bulk soil (Fig. S6). In contrast, we found very different patterns at almost every level of taxonomy in the fungi, which indicated even greater variation in the fungal microbiota than in the bacterial microbiota when comparing communities in the rhizosphere with those in the bulk soil (Figs. S2–S6).

**Table 2:** OTU clustering of sequence data. R-1 to R-9 are samples collected from the rhizosphere. S-1 to S-9 are samples collected from the bulk soil. Taxon reads: annotated clean reads that are used to set up OTUs. Unclassified reads: reads without annotations. Singletons: single reads that could not be clustered to any OTUs (these reads were not subjected to further analysis).

**18 samples from 3 sites**		**16S**					**ITS**				
		**Clean reads**	**Taxon reads**	**Unclassified**	**Singletons**	**OTUs**	**Clean reads**	**Taxon reads**	**Unclassified**	**Singletons**	**OTUs**
**Site 1**	R-1	38,089	30,857	204	7,028	2,332	43,394	42,353	0	1,041	452
	R-2	34,371	25,898	150	8,323	2,216	38,815	38,013	0	802	340
	R-3	37,691	30,274	216	7,201	2,433	39,207	38,570	0	637	327
	S-1	39,050	25,265	789	12,996	3,001	42,005	40,290	0	1,715	734
	S-2	34,300	22,183	768	11,349	2,684	40,097	38,667	0	1,430	531
	S-3	37,364	29,709	930	6,725	3,241	35,942	34,819	0	1,123	564
**Site 2**	R-4	30,760	22,398	357	8,005	2,799	39,959	38,325	0	1,634	474
	R-5	36,680	25,620	434	10,626	2,999	40,274	38,028	0	2,246	552
	R-6	38,426	28,191	429	9,806	3,000	42,656	41,632	0	1,024	532
	S-4	39,884	30,053	1,206	8,625	3,190	38,430	36,290	0	2,140	340
	S-5	37,796	26,394	993	10,409	3,018	36,392	34,976	0	1,416	426
	S-6	36,649	25,629	1,344	9,676	2,966	41,628	40,503	0	1,125	462
**Site 3**	R-7	33,739	24,797	243	8,699	2,695	42,305	41,409	0	896	556
	R-8	34,748	25,363	323	9,062	2,795	39,722	38,696	0	1,026	466
	R-9	34,434	24,903	199	9,332	2,612	37,212	36,505	0	707	412
	S-7	37,973	30,256	1,777	5,940	2,837	41,156	40,657	0	499	244
	S-8	38,061	27,511	1,366	9,184	3,014	35,699	35,165	0	534	144
	S-9	38,837	27,550	1,795	9,492	2,987	42,364	41,909	0	455	199

### Alpha and beta diversity analysis of samples

**Figure 2:**
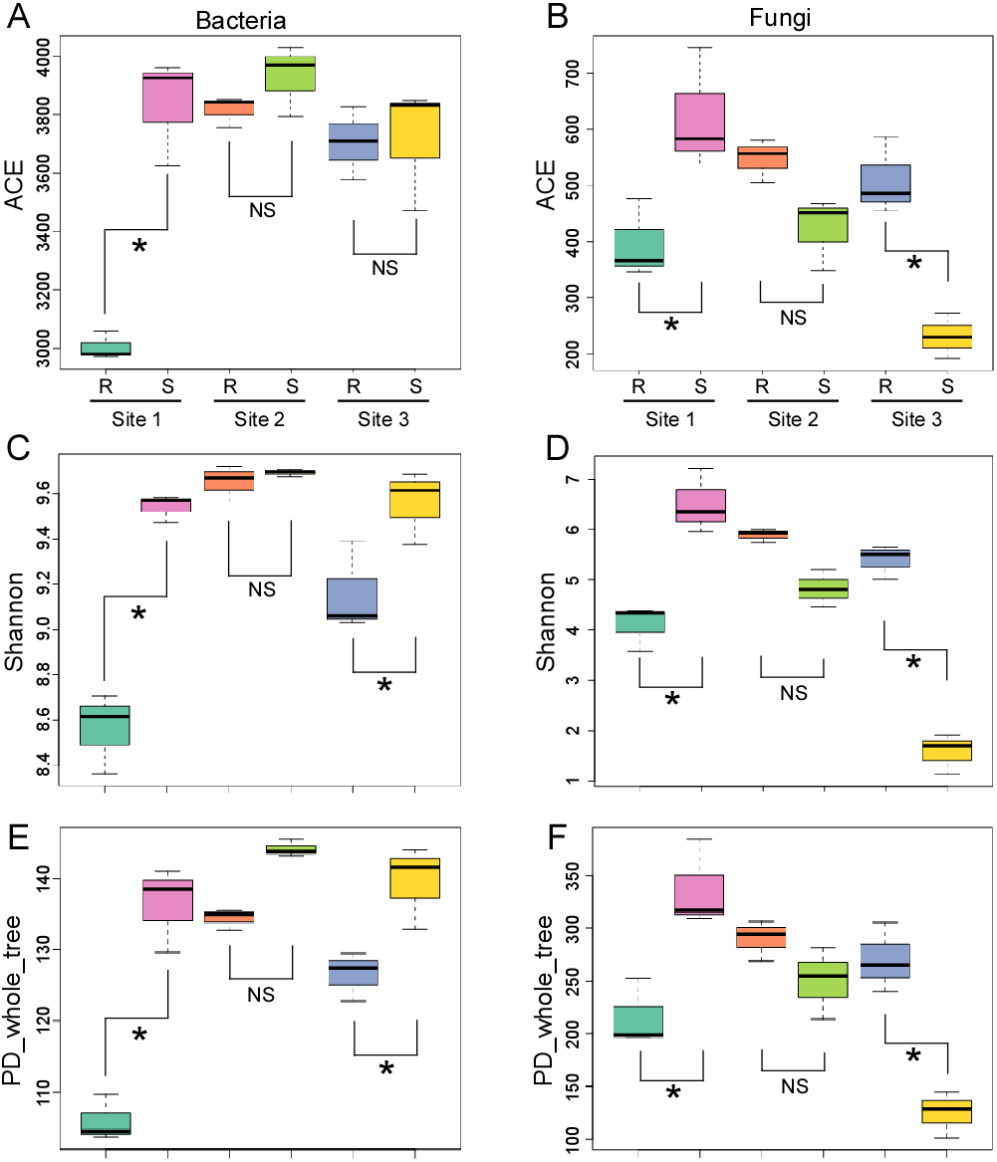
Alpha diversity of the bacterial and fungal communities. (A), (C) and (E), three indices for bacteria. (B), (D) and (F), three indices for fungi. Alpha diversity estimates represent three biological replicates for the rhizosphere (R) and the bulk soil (S) from the three sites. Significant changes (*P* < 0.05), determined by the Tukey test, are marked by an asterisk. NS, not significant.

To quantify alpha diversity, the diversities of bacteria and fungi in each sample were analyzed based on the diversity indices of ACE, Shannon, PD_whole_tree and Good’s_coverage. The Good’s_coverage index for all the samples was greater than 0.95 for bacteria (Table S4) and greater than 0.99 for fungi (Table S5), demonstrating that the sequencing depth was acceptable. For bacteria, generally, the three indices of ACE, Shannon and PD_whole_tree were lower in the rhizosphere soil compared to that in the bulk soil. Specifically, all three indices were significantly different in site 1, with similar variation tendencies in sites 2 and 3 (Fig. 2). This demonstrated the selective effect on bacterial diversity of the rhizosphere. In contrast, the differences in these indices between rhizosphere and bulk soil were different or even opposite in different sites for the fungi. For example, all three indices decreased significantly in the rhizosphere soil from site 1, but increased significantly in the rhizosphere soil of site 3 compared to that in the corresponding bulk soil (Fig. 2). These results indicated that the effects of the ginkgo rhizosphere on the distribution of the bacteria may be different from that of the fungi.

**Figure 3:**
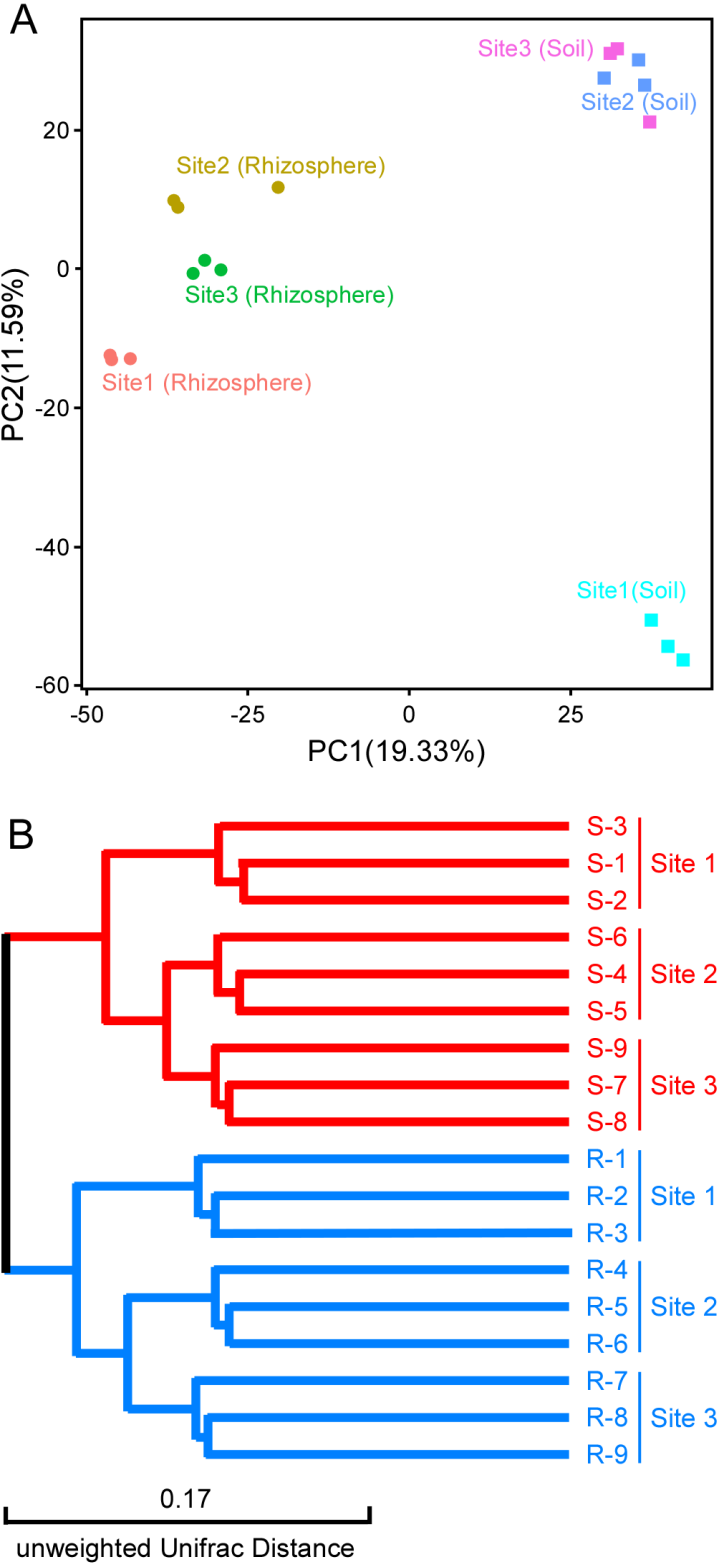
Beta diversity of the bacterial communities. (A) PCA analysis. Samples from rhizosphere and control soil are marked. (B) UPGMA cluster analysis. Samples collected from rhizosphere are marked in blue, and samples collected from the bulk soil are marked in red.

For beta diversity, PCA analysis was carried out to cluster bacterial and fungal communities in rhizosphere and bulk soil, according to the different sampling sites. At the OTU level, PC1 explained 19.33% and PC2 11.59% of the total variation in bacteria, and the rhizosphere samples were clearly distinguishable from the bulk soil samples (Fig. 3A). In contrast, PC1 explained 13.71% and PC2 10.05% of the total variation in fungi; samples from the rhizosphere and bulk soil did not separate clearly with respect to the fungi (Fig. S7A). We also performed UPGMA cluster analysis and built cluster trees for the samples. Using this approach, all the samples collected from the rhizosphere were distinguishable from the samples from the bulk soil with respect to bacteria, while the situation was less clear-cut with respect to the fungi (Fig. 3B, Fig. S7B).

### Variations in the microbiota in the ginkgo rhizosphere at the genus level

**Figure 4:**
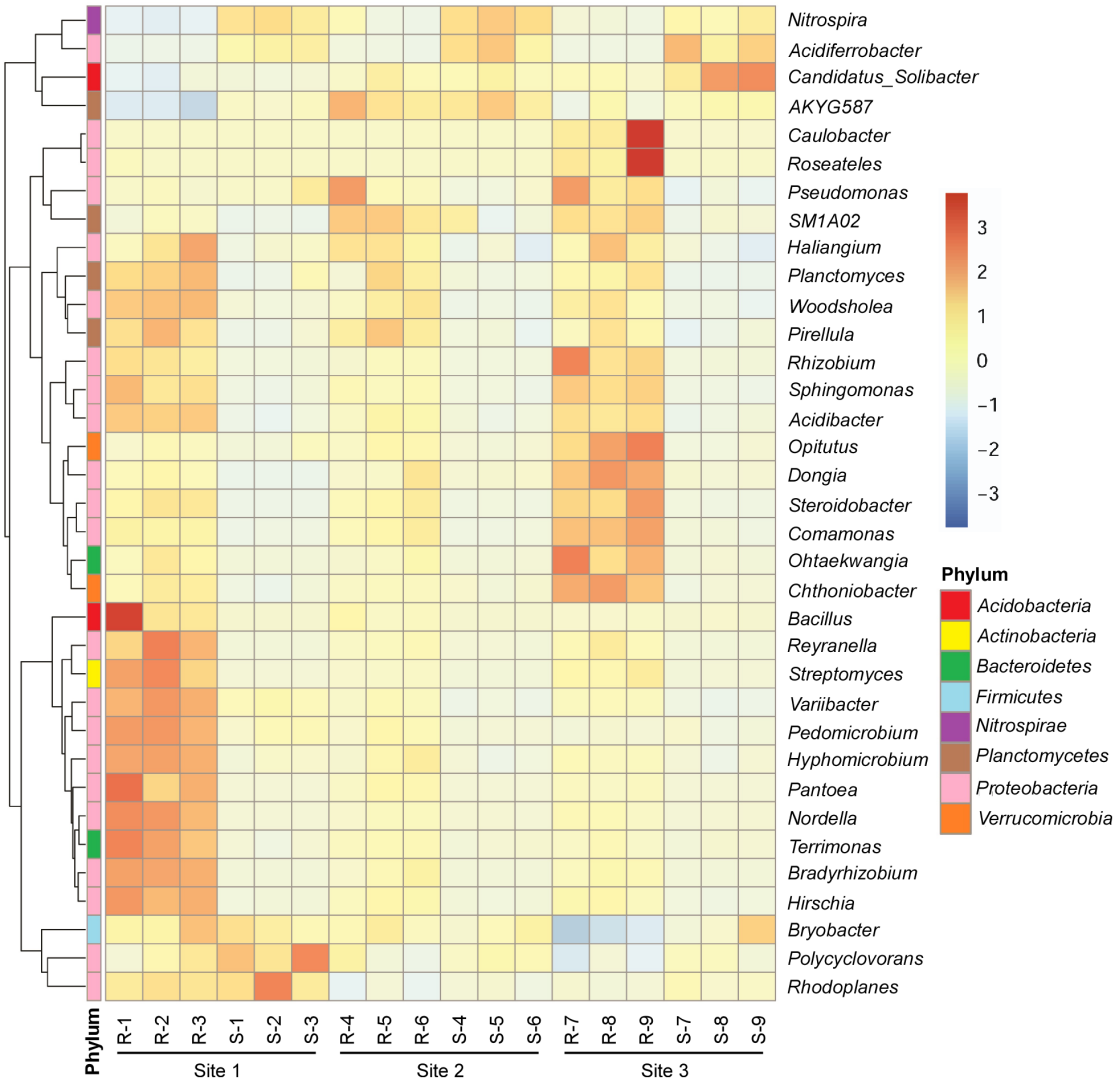
The abundance of the 35 most-abundant genera of bacteria in the rhizosphere and the bulk soil from the three sites. The taxonomies of genera to the level of phylum are marked by different colors. The relative abundances of the 35 genera are scaled by a Z-score color gradient bar. The red colored data represent genera that have higher abundance than average. The blue colored data represent genera that have lower abundance than average. A Z-score of 0 represents a genus abundance value that is equal to the average abundance value.

To take a closer look at the variation in the microbiota of the ginkgo rhizosphere, the 35 most abundant genera of bacteria were compared between the rhizosphere and the bulk soil. Interestingly, the abundance of most of the genera varied between the rhizosphere and the bulk soil (Fig. 4). Several genera showed increased abundance in the rhizosphere, including *Bradyrhizobium*, *Rhizobium*, *Sphingomonas* and *Streptomyces*. In contrast, there were also some genera which showed decreased abundance in the rhizosphere compared to that in the bulk soil, including *Nitrospira* (Fig. 4). We also examined the relative abundance values in the rhizosphere and bulk soil of the 35 highest-frequency fungal genera, and found that, although some genera showed significantly different abundances between rhizosphere and bulk soil at one collection site, this was not consistent across the other sites (Fig. S8). This indicated that, for individual fungal genera, the selective effect (positive or negative) of the rhizosphere was not significant as it was in bacteria.

### Correlation and KEGG functional analysis in bacteria

Based on the changes in abundance of different genera in different soil samples, the relationships between the abundance values of the various bacterial genera were determined. It was obvious that *Rhizobium* and *Pantoea* dominated the microbiota. *Rhizobium* was the most abundant genus, and had a significant positive relationship with *Bosea*. *Pantoea* was also highly abundant and had very close relationships with many other genera, including *Bradyrhizobium* (Fig. S9). The relationship network of these genera indicated a complex functional collaboration within the microbiota. To analyze the functional diversities of bacteria, the KEGG functional enrichment analysis of bacterial microbiota was compared between the rhizosphere and the bulk soil. The frequency of ATP-binding cassette (ABC) transporters was enriched significantly in the rhizosphere while the frequency of the two-component system decreased significantly in the rhizosphere (Fig. S10). This indicated a functional divergence of bacterial microbiota in response to the rhizosphere of ginkgo roots.

## Discussion

With the development of the ginkgo-based pharmaceutical industry and of ginkgo horticulture, it is increasingly important to fully understand the different aspects of ginkgo biology, including the relationship with its root microbiota. Most of the previous research on ginkgo had focused on the biosynthesis pathways of various bioactive compounds present in ginkgo leaves, which are raw materials for the pharmaceutical industry. On the other hand, little research has been carried out on ginkgo roots. To our knowledge, this research represents the first report on the relationship between ginkgo roots and the soil microbiota.

In this study, the 16S rDNA in bacteria and the ITS rDNA sequences in fungi were amplified and sequenced. We did not find the contaminant sequences from chloroplast, mitochondrial or nuclear DNA, which have frequently occurred in related research (Beckers et al., 2016). This finding showed that our PCR approach was optimized and suitable for this research. Considering the complex nature of the soil environment, which may cause changes in microbiota composition, we chose three different and independent sites for sample collection. Only those changes which occurred in all three sites were discussed.

One of the important findings in this study was the accumulation in the rhizosphere of species of *Rhizobium* and its fellow rhizobial genus *Bradyrhizobium*. *Rhizobium* and *Bradyrhizobium* are nitrogen-fixing bacteria which induce the development of nodules in the roots of legume plant hosts (Long, 1996). In addition, flavonoids are widely accepted to be regulators of symbiotic interactions, acting as specific signals between plant hosts and *Rhizobium* (Mierziak, Kostyn & Kulma, 2014). Considering that flavonoids are one of the most important groups of secondary plant metabolites in ginkgo (Kleijnen & Knipschild, 1992), it is possible that ginkgo root cells secrete flavonoids, which act as specific signals to attract the accumulation of *Rhizobium* and *Bradyrhizobium* in the rhizosphere. However, despite the accumulation of *Rhizobium* and *Bradyrhizobium* in the rhizosphere, we did not find any nodules on ginkgo roots. This might be due to the lack of Nod factor receptors or to defects of the subsequent kinase cascade in ginkgo, which is crucial to nodule formation in legumes (Gage, 2004; Smit et al., 2007). Following the recent publication of the draft genome of *G. biloba* (Guan et al., 2016), it would be interesting to identify the missing steps in ginkgo which are associated with its inability to form nodules.

We also observed an accumulation of *Sphingomonas* in the ginkgo rhizosphere. Bacteria within the genus *Sphingomonas* share the common capacity to degrade a broad range of aromatic compounds (Fredrickson et al., 1995). Thus, the accumulation of *Sphingomonas* in the rhizosphere indirectly suggested the secretion of different aromatic secondary metabolites from ginkgo roots, which attracted the accumulation of aromatics-consuming *Sphingomonas* species.

*Streptomyces* is the largest genus of the Actinobacteria, with more than 500 species having been described (Euzeby, 2008). *Streptomyces* not only produces a volatile metabolite, geosmin, which result in the distinct “earthy” odor of soil, but also produces antibiotics, which they use to compete with other bacteria for resources. A number of them have been developed as antifungals, antibiotics and chemotherapeutic drugs to improve human health (Raja & Prabakarana, 2011). The benefits to ginkgo of *Streptomyces* accumulation in the rhizosphere are currently unknown, but this phenomenon could increase the complexity of the composition of the microbiota in the rhizosphere.

In contrast to those bacterial genera which accumulated in the ginkgo rhizosphere, there were also some genera which decreased in the rhizosphere compared to the bulk soil. The genus *Nitrospira* consists of a group of species which are widely distributed in many natural environments (Bartosch et al., 2002), and they are considered to play important roles in the nitrogen cycle in both water and soil (Hayatsu, Tago & Saito, 2008). Despite the potential advantage of an exogenous nitrate supply to ginkgo, *Nitrospira* did not accumulate in the rhizosphere but rather was present at lower frequencies in the rhizosphere than in the bulk soil. This decline may due to the inhibitory effects of antibiotics produced by other rhizobacteria (*Streptomyces,* for instance), which accumulated in the rhizosphere. Alternatively, the decline may be caused by the complex secondary metabolites secreted by ginkgo roots. It has been reported that flavonoids, secreted by root cells, had both positive and negative effects on nodule formation by nitrogen-fixing bacteria (Cooper, 2004; Khandual, 2007). Thus, it is possible that certain compounds secreted by ginkgo root cells prevented the accumulation of certain bacteria, including *Nitrospira*.

ABC transporters are integral membrane proteins that couple the transport of substrates across lipid bilayers to the hydrolysis of ATP (Hollenstein, Dawson & Locher, 2007). In bacteria, ABC transporters are important factors catalyzing the uptake of nutrients and the efflux of toxic or antimicrobial agents, which are crucial for bacterial survival (Davidson & Chen, 2004). The accumulation in the rhizosphere of bacteria enriched with respect to ABC transporters is consistent with the function of the rhizosphere, which mediates the exchange of inorganic and organic substances between the roots and the soil (Ryan, Delhaize & Jones, 2001). Two-component systems are characterized by a sensor kinase consisting of a signal-recognition domain with unique specificity, coupled to an auto-kinase domain, and they are the major means by which bacteria recognize and respond to a range of environmental stimuli (Hoch, 2000). For the bacteria in the rhizosphere, the frequency of two-component systems decreased compared to that in the bulk soil. We propose that bacteria must cope with different environmental stimuli in the soil. For the bacteria in the rhizosphere, the microenvironment is greatly affected by the plants. These bacteria are partially “protected” by the rhizosphere, and there is no need for these bacteria to employ numerous two-component systems to cope with the different challenges from the ever- changing environment as would be the case for the bacteria in the bulk soil.

Compared with the clear changes in bacterial frequency between the rhizosphere and bulk soil, the responses of fungal frequency between the two soil types varied between the different collection sites. Many fungal genera accumulated in the rhizosphere of one collection site, yet decreased at other collection sites (Fig. S8). Considering the different and complex subcellular structures of fungi, it is possible that the substances secreted by root cells and bacteria have relatively little effect on the distribution of the fungal microbiota.

## Conclusions

In this research, the structural and functional diversities of microbiota between the *Ginkgo biloba* root rhizosphere and the corresponding bulk soil were investigated. A number of bacterial genera showed significantly different abundance in the rhizosphere compared to the bulk soil, including *Bradyrhizobium*, *Rhizobium*, *Sphingomonas*, *Streptomyces* and *Nitrospira*. Functional enrichment analysis of bacterial microbiota revealed consistently increased abundance of ATP-binding cassette (ABC) transporters and decreased abundance of two-component systems in the rhizosphere community, compared to the bulk soil community. In contrast, the situation was more complex and inconsistent for fungi, indicating the independency of the rhizosphere fungal community on the local microenvironment. This study was the first attempt to characterize the microbiota in the ginkgo rhizosphere, which indicated a complex relationship between ginkgo and the microbial communities in the soil.

##  Supplemental Information

10.7717/peerj.8051/supp-1 Figure S1 Distribution of *Ginkgo biloba* in China **(A)** Number of cities in each province in China, where *G. biloba* is treated as a municipal tree. Data were obtained through website searches. **(B)** A common street in the city of Hangzhou. Ginkgo trees are planted along both sides of the street.

10.7717/peerj.8051/supp-2 Figure S2 Relative abundance of ten most-abundant phyla **(A)** Bacteria. **(B)**Fungi.

10.7717/peerj.8051/supp-3 Figure S3 Relative abundance of the ten most-abundant classes **(A)** Bacteria. **(B)**Fungi.

10.7717/peerj.8051/supp-4 Figure S4 Relative abundance of the ten most-abundant orders **(A)** Bacteria. **(B)**Fungi.

10.7717/peerj.8051/supp-5 Figure S5 Relative abundance of the ten most-abundant families **(A)** Bacteria. **(B)**Fungi.

10.7717/peerj.8051/supp-6 Figure S6 Relative abundance of the ten most-abundant genera **(A)** Bacteria. **(B)**Fungi.

10.7717/peerj.8051/supp-7 Figure S7 Beta diversity of the fungal communities **(A)** PCA analysis. Samples from the rhizosphere and the bulk soil are marked. **(B)** UPGMA cluster analysis. Samples collected from the rhizosphere are marked in blue, and samples collected from the bulk soil are marked in red.

10.7717/peerj.8051/supp-8 Figure S8 The abundance of the 35 most-abundant genera of fungi in the rhizosphere and the bulk soil from the threesites The taxonomies of genera to the level of phylum are marked by different colors. The relative abundances of the 35 genera are scaled by a Z-score color gradient bar. The red colored data represent genera that have higher abundance than average. The blue colored data represent genera that have lower abundance than average. A Z-score of 0 represents a genus abundance value that is equal to the average abundance value.

10.7717/peerj.8051/supp-9 Figure S9 Correlation analysis in bacteria Each point represents a genus. The size and color of the spots represent the abundance of the genera. The relationships among correlated genera are represented by the size and color of lines.

10.7717/peerj.8051/supp-10 Figure S10 KEGG functional analysis in bacteria Bars in the left, mean values of KEGG enrichment. Bars across circles, 95% confidence interval of the differences. Center of circles, differences of the mean values. Numbers in the right, p values of the significance test.

10.7717/peerj.8051/supp-11 Table S1 The name of cities treating *Ginkgo biloba* as a municipaltree

10.7717/peerj.8051/supp-12 Table S2 Numbers of bacterial taxon tags at different levels of taxonomy

10.7717/peerj.8051/supp-13 Table S3 Numbers of fungal taxon tags at different levels of taxonomy

10.7717/peerj.8051/supp-14 Table S4 Alpha diversity analysis of the bacterial communities

10.7717/peerj.8051/supp-15 Table S5 Alpha diversity analysis of the fungal communities

## Additional Information and Declarations

### Data Availability

The following information was supplied regarding data availability: Data is available at NCBI SRA. Bacteria: PRJNA565829, Fungi: PRJNA566252.

## References

[ref-1] Bachy C, Dolan JR, Lopez-Garcia P, Deschamps P, Moreira D (2013). Accuracy of protist diversity assessments: morphology compared with cloning and direct pyrosequencing of 18S rRNA genes and ITS regions using the conspicuous tintinnid ciliates as a case study. ISME Journal.

[ref-2] Bartosch S, Hartwig C, Spieck E, Bock E (2002). Immunological detection of Nitrospira-like bacteria in various soils. Microbial Ecology.

[ref-3] Beckers B, Op De Beeck M, Thijs S, Truyens S, Weyens N, Boerjan W, Vangronsveld J (2016). Performance of 16s rDNA primer pairs in the study of rhizosphere and endosphere bacterial microbiomes in metabarcoding studies. Frontiers in Microbiology.

[ref-4] Bengtsson-Palme J, Ryberg M, Hartmann M, Branco S, Wang Z, Godhe A, Wit P, Sánchez-García M, Ebersberger I, Sousa F (2013). Improved software detection and extraction of ITS1 and ITS2 from ribosomal ITS sequences of fungi and other eukaryotes for analysis of environmental sequencing data. Methods in Ecology and Evolution.

[ref-5] Berendsen RL, Pieterse CMJ, Bakker PAHM (2012). The rhizosphere microbiome and plant health. Trends in Plant Science.

[ref-6] Berg G, Grube M, Schloter M, Smalla K (2014). Unraveling the plant microbiome: looking back and future perspectives. Frontiers in Microbiology.

[ref-7] Bokulich NA, Subramanian S, Faith JJ, Gevers D, Gordon JI, Knight R, Mills DA, Caporaso JG (2013). Quality-filtering vastly improves diversity estimates from Illumina amplicon sequencing. Nature Methods.

[ref-8] Caporaso JG, Kuczynski J, Stombaugh J, Bittinger K, Bushman FD, Costello EK, Fierer N, Pena AG, Goodrich JK, Gordon JI, Huttley GA, Kelley ST, Knights D, Koenig JE, Ley RE, Lozupone CA, McDonald D, Muegge BD, Pirrung M, Reeder J, Sevinsky JR, Turnbaugh PJ, Walters WA, Widmann J, Yatsunenko T, Zaneveld J, Knight R (2010). QIIME allows analysis of high-throughput community sequencing data. Nature Methods.

[ref-9] Caporaso JG, Lauber CL, Walters WA, Berg-Lyons D, Lozupone CA, Turnbaugh PJ, Fierer N, Knight R (2011). Global patterns of 16S rRNA diversity at a depth of millions of sequences per sample. Proceedings of the National Academy of Sciences of the United States of America.

[ref-10] Chisholm ST, Coaker G, Day B, Staskawicz BJ (2006). Host-microbe interactions: shaping the evolution of the plant immune response. Cell.

[ref-11] Cooper JE (2004). Multiple responses of rhizobia to flavonoids during legume root infection. Advances in botanical research.

[ref-12] Davidson AL, Chen J (2004). ATP-binding cassette transporters in bacteria. Annual Review of Biochemistry.

[ref-13] Edgar RC (2013). UPARSE: highly accurate OTU sequences from microbial amplicon reads. Nature Methods.

[ref-14] Edgar RC, Haas BJ, Clemente JC, Quince C, Knight R (2011). UCHIME improves sensitivity and speed of chimera detection. Bioinformatics.

[ref-15] Euzeby J (2008). Genus streptomyces. List of Prokaryotic names with standing in nomenclature.

[ref-16] Fredrickson J, Balkwill D, Drake G, Romine M, Ringelberg D, White D (1995). Aromatic-degrading Sphingomonas isolates from the deep subsurface. Applied and Environmental Microbiology.

[ref-17] Gage DJ (2004). Infection and invasion of roots by symbiotic, nitrogen-fixing rhizobia during nodulation of temperate legumes. Microbiology and Molecular Biology Reviews.

[ref-18] Guan R, Zhao Y, Zhang H, Fan G, Liu X, Zhou W, Shi C, Wang J, Liu W, Liang X, Fu Y, Ma K, Zhao L, Zhang F, Lu Z, Lee SM, Xu X, Yang H, Fu C, Ge S, Chen W (2016). Draft genome of the living fossil Ginkgo biloba. Gigascience.

[ref-19] Haas BJ, Gevers D, Earl AM, Feldgarden M, Ward DV, Giannoukos G, Ciulla D, Tabbaa D, Highlander SK, Sodergren E, Methe B, DeSantis TZ, Petrosino JF, Knight R, Birren BW (2011). Chimeric 16S rRNA sequence formation and detection in Sanger and 454-pyrosequenced PCR amplicons. Genome Research.

[ref-20] Hartmann A, Rothballer M, Schmid M (2008). Lorenz Hiltner, a pioneer in rhizosphere microbial ecology and soil bacteriology research. Plant and Soil.

[ref-21] Hayatsu M, Tago K, Saito M (2008). Various players in the nitrogen cycle: diversity and functions of the microorganisms involved in nitrification and denitrification. Soil Science & Plant Nutrition.

[ref-22] Hoch JA (2000). Two-component and phosphorelay signal transduction. Current Opinion in Microbiology.

[ref-23] Hollenstein K, Dawson RJP, Locher KP (2007). Structure and mechanism of ABC transporter proteins. Current Opinion in Structural Biology.

[ref-24] Khandual S (2007). Flavonoids as signaling molecules and regulators of root nodule development. Dynamic Soil, Dynamic Plant.

[ref-25] Kleijnen J, Knipschild P (1992). Ginkgo biloba. The Lancet.

[ref-26] Klindworth A, Pruesse E, Schweer T, Peplies J, Quast C, Horn M, Glöckner FO (2012). Evaluation of general 16S ribosomal RNA gene PCR primers for classical and next-generation sequencing-based diversity studies. Nucleic Acids Research.

[ref-27] Long SR (1996). Rhizobium symbiosis: nod factors in perspective. The Plant Cell.

[ref-28] Magoc T, Salzberg SL (2011). FLASH: fast length adjustment of short reads to improve genome assemblies. Bioinformatics.

[ref-29] Mierziak J, Kostyn K, Kulma A (2014). Flavonoids as important molecules of plant interactions with the environment. Molecules.

[ref-30] Ni J, Dong L, Jiang Z, Yang X, Chen Z, Wu Y, Xu M (2018a). Comprehensive transcriptome analysis and flavonoid profiling of Ginkgo leaves reveals flavonoid content alterations in day–night cycles. PLOS ONE.

[ref-31] Ni J, Dong L, Jiang Z, Yang X, Sun Z, Li J, Wu Y, Xu M (2018b). Salicylic acid-induced flavonoid accumulation in Ginkgo biloba leaves is dependent on red and far-red light. Industrial Crops and Products.

[ref-32] Ni J, Hao J, Jiang Z, Zhan X, Dong L, Yang X, Sun Z, Xu W, Wang Z, Xu M (2017). NaCl induces flavonoid biosynthesis through a putative novel pathway in post-harvest Ginkgo leaves. Frontiers in Plant Science.

[ref-33] R Core Team (2015). https://www.R-project.org/.

[ref-34] Raja A, Prabakarana P (2011). Actinomycetes and drug-an overview. American Journal of Drug Discovery and Development.

[ref-35] Rimbach G, Gohil K, Matsugo S, Moini H, Saliou C, Virgili F, Weber SU, Packer L (2001). Induction of glutathione synthesis in human keratinocytes by Ginkgo biloba extract (EGb761). Biofactors.

[ref-36] Ryan P, Delhaize E, Jones D (2001). Function and mechanism of organic anion exudation from plant roots. Annual Review of Plant Physiology and Plant Molecular Biology.

[ref-37] Smit P, Limpens E, Geurts R, Fedorova E, Dolgikh E, Gough C, Bisseling T (2007). Medicago LYK3, an entry receptor in rhizobial nodulation factor signaling. Plant Physiology.

[ref-38] Sreenivasaprasad S, Sharada K, Brown AE, Mills PR (1996). PCR-based detection of Colletotrichum acutatum on strawberry. Plant Pathology.

[ref-39] Turner TR, James EK, Poole PS (2013). The plant microbiome. Genome Biology.

[ref-40] Youssef N, Sheik CS, Krumholz LR, Najar FZ, Roe BA, Elshahed MS (2009). Comparison of species richness estimates obtained using nearly complete fragments and simulated pyrosequencing-generated fragments in 16S rRNA gene-based environmental surveys. Applied and Environmental Microbiology.

[ref-41] Zhou Z-Y (2009). An overview of fossil Ginkgoales. Palaeoworld.

