# Supplementary material for: High-throughput sequence analysis reveals variation in the relative abundance of components of the bacterial and fungal microbiota in the rhizosphere of Ginkgo biloba
Source: PeerJ. 2019 Nov 15;7:e8051. doi: 10.7717/peerj.8051 (PMC6859886; doi:10.7717/peerj.8051)
Supplement: Figure S1 — (A) Number of cities in each province in China, where G. biloba is treated as a municipal tree. Data were obtained through website searches. (B) A common street in the city of Hangzhou. Ginkgo trees are planted along both sides of the street. [file peerj-07-8051-s001.pdf]

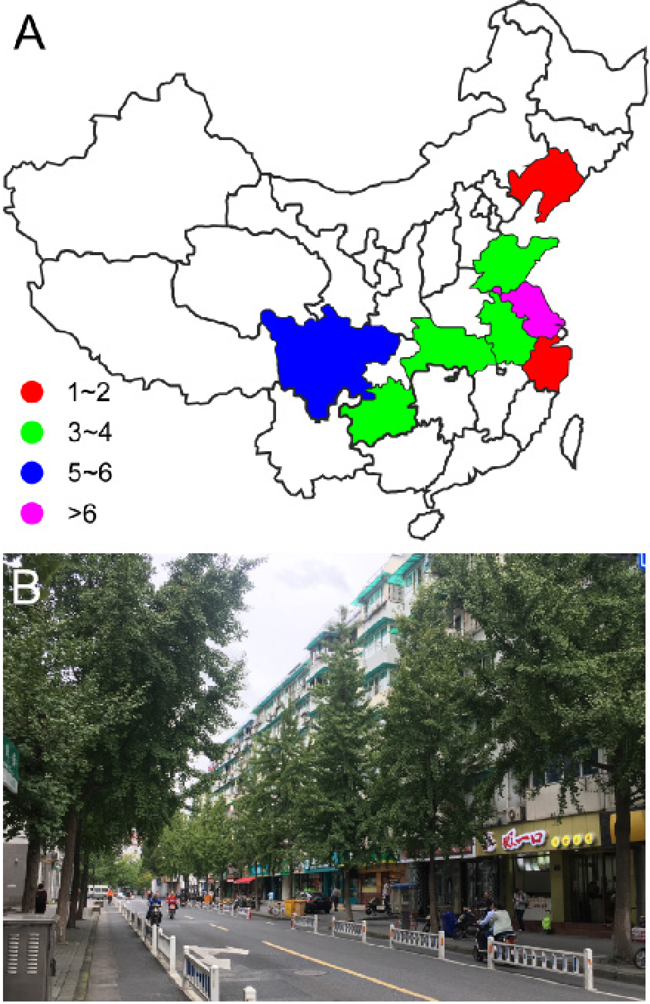

Figure S1. Distribution of *Ginkgo biloba* in China. (A) Number of cities in each province in China, where *G. biloba* is treated as a municipal tree. Data were obtained through website searches. (B) A common street in the city of Hangzhou. Ginkgo trees are planted along both sides of the street.
