# Supplementary figures and images for: High-throughput sequence analysis reveals variation in the relative abundance of components of the bacterial and fungal microbiota in the rhizosphere of Ginkgo biloba
Source: PeerJ. 2019 Nov 15;7:e8051. doi: 10.7717/peerj.8051 (PMC6859886; doi:10.7717/peerj.8051)

**A**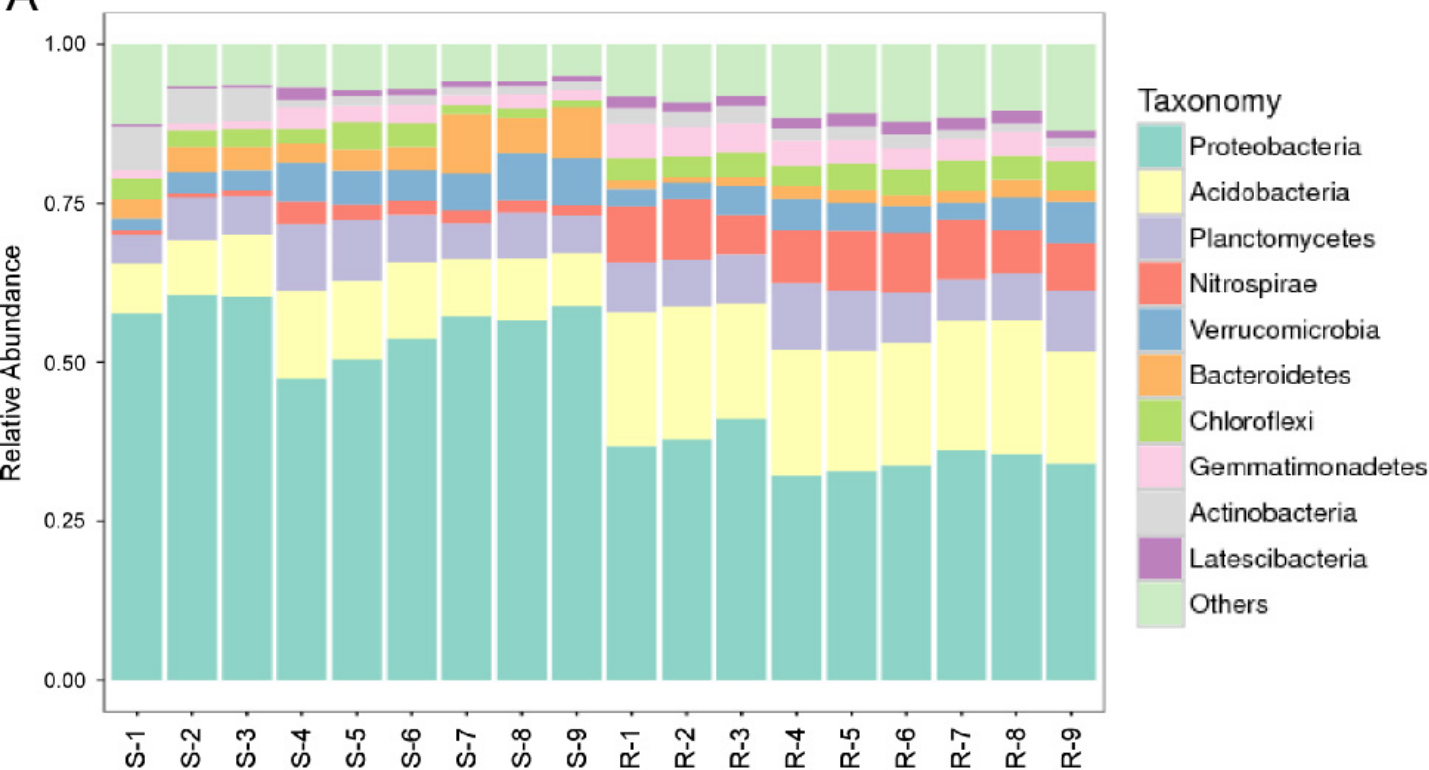**B**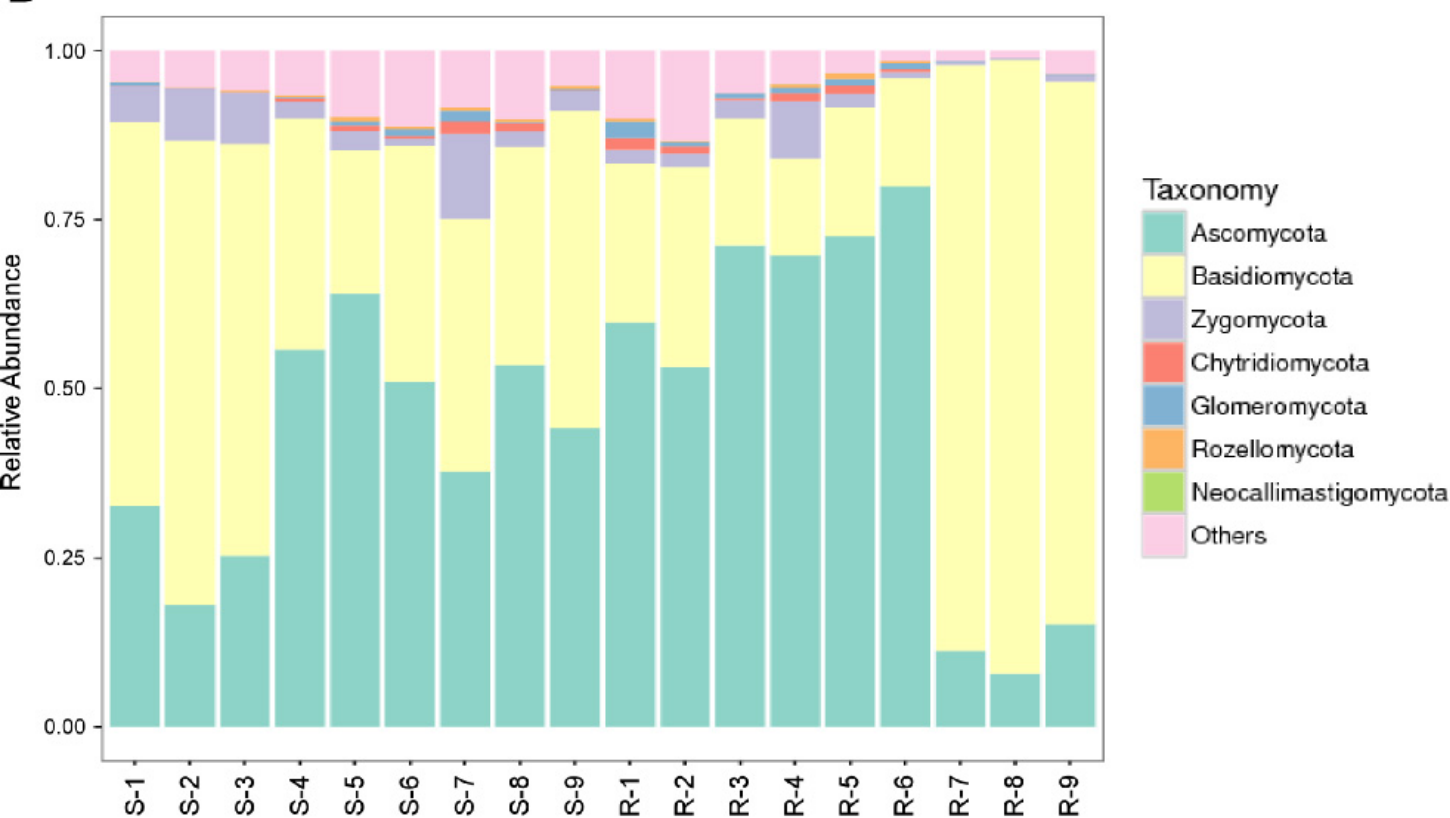

**Figure S2. Relative abundance of ten most-abundant phyla. (A) Bacteria. (B) Fungi.**

Supplement: Figure S2 — (A) Bacteria. (B)Fungi. [file peerj-07-8051-s002.pdf]

**A**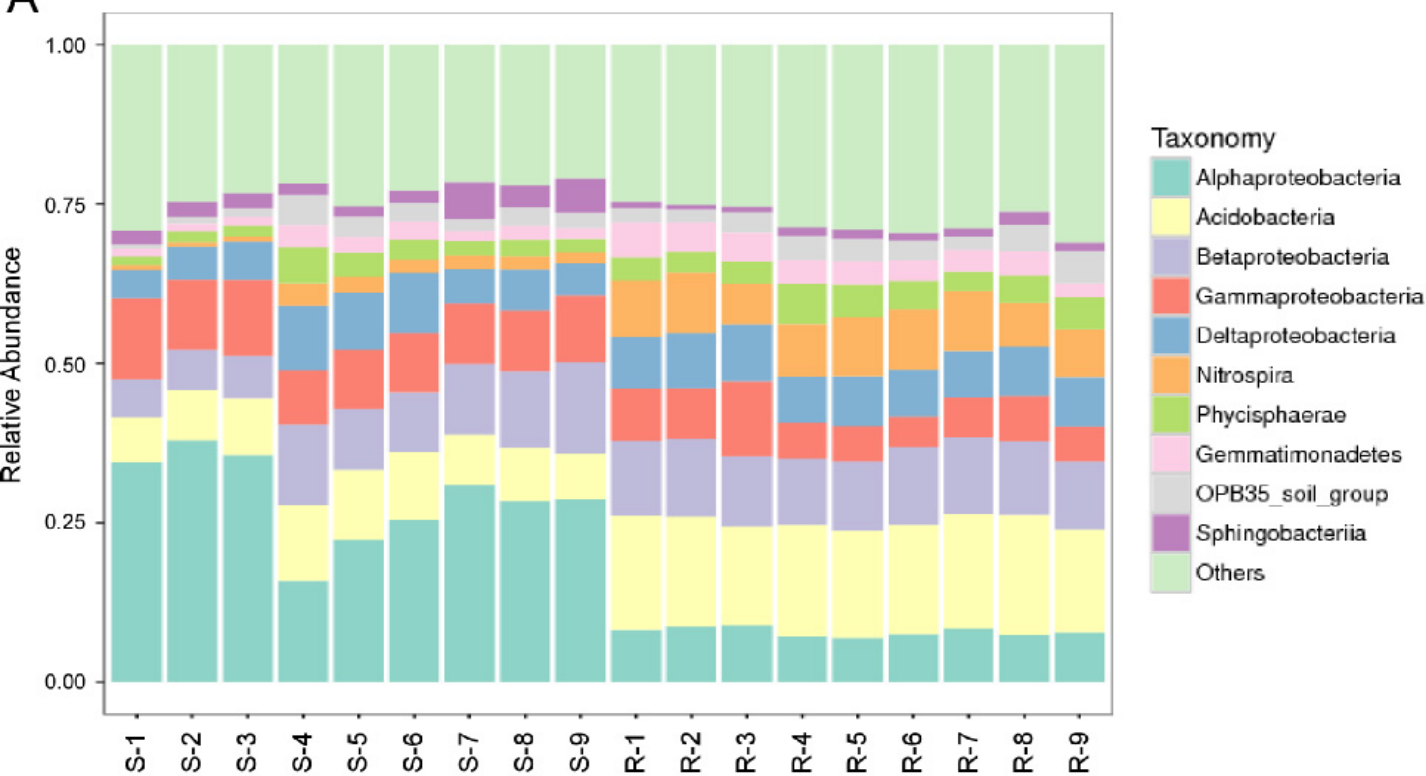**B**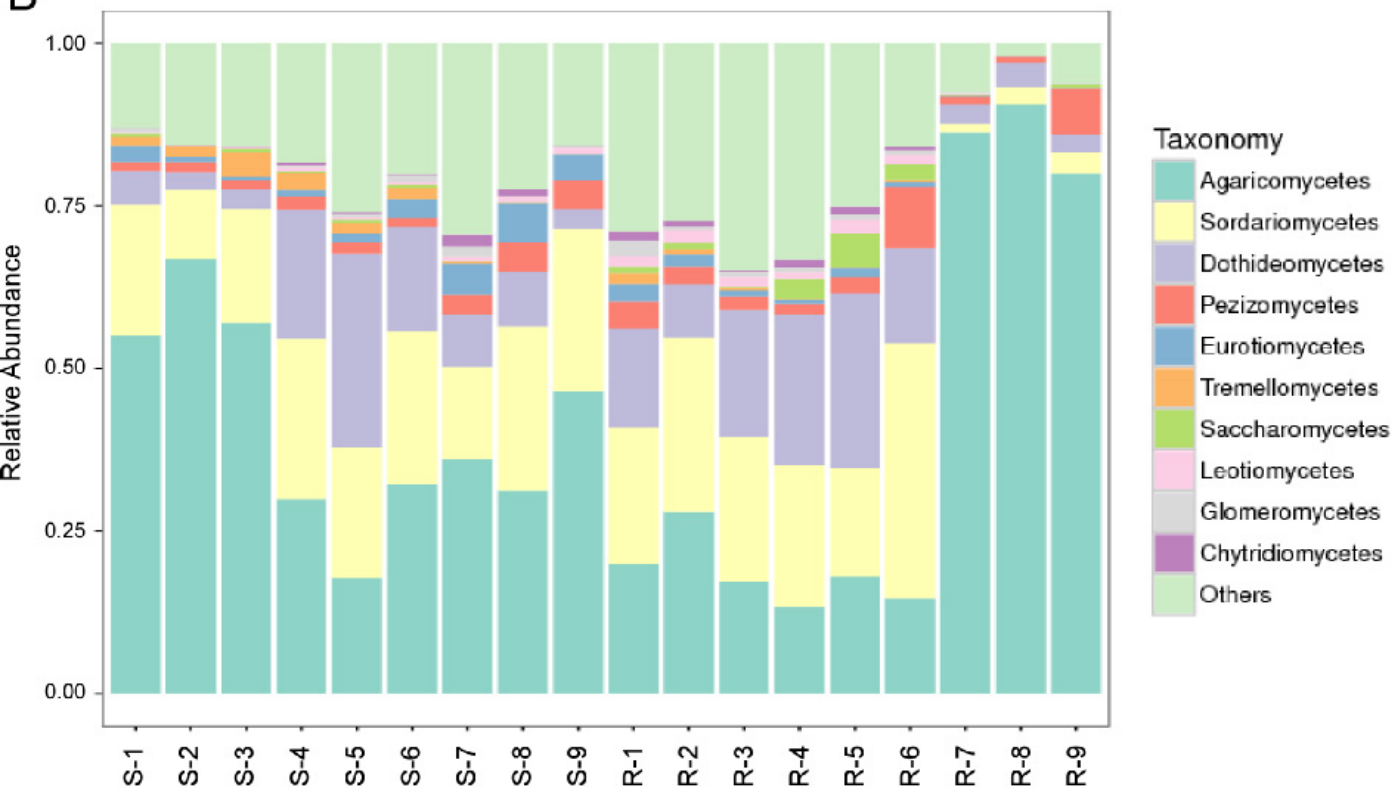

**Figure S3. Relative abundance of the ten most-abundant classes. (A) Bacteria. (B) Fungi.**

Supplement: Figure S3 — (A) Bacteria. (B)Fungi. [file peerj-07-8051-s003.pdf]

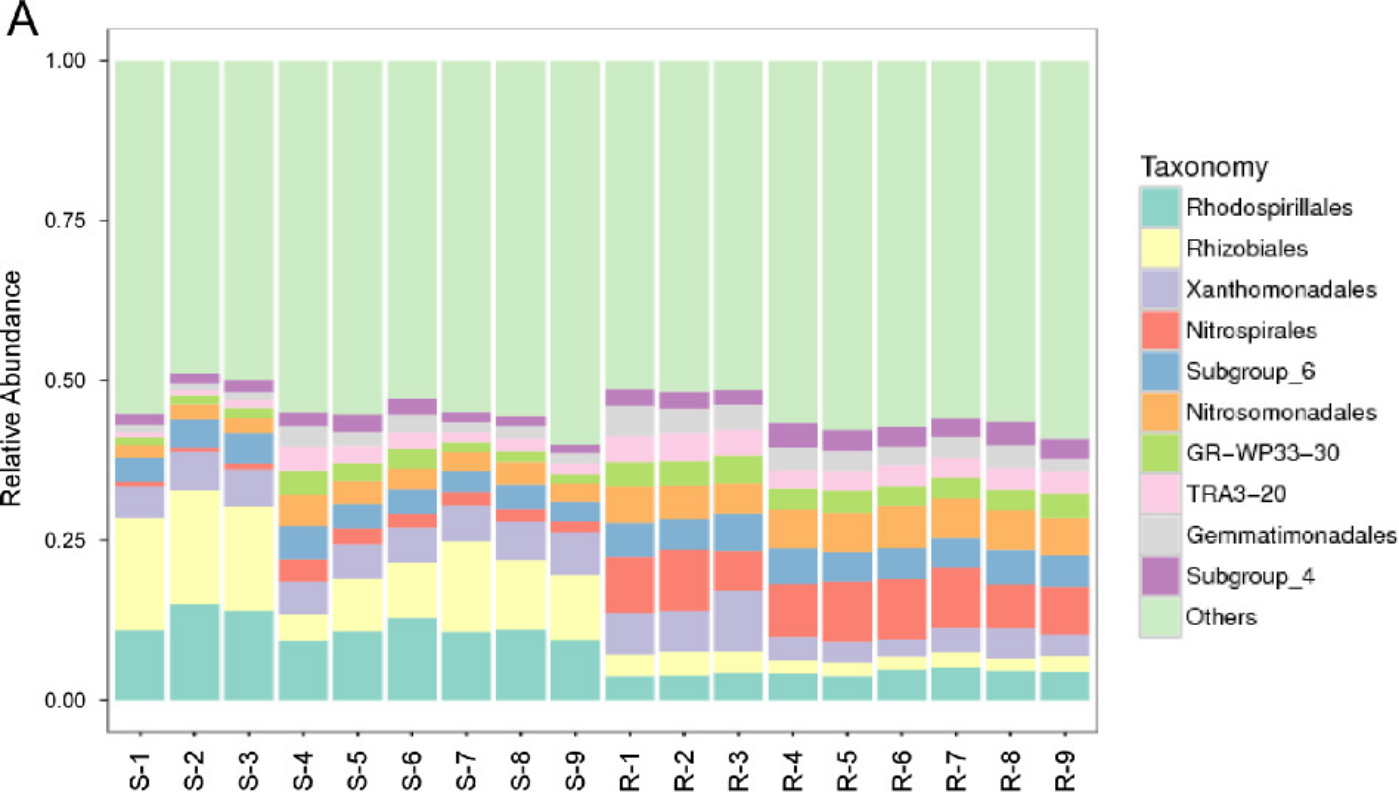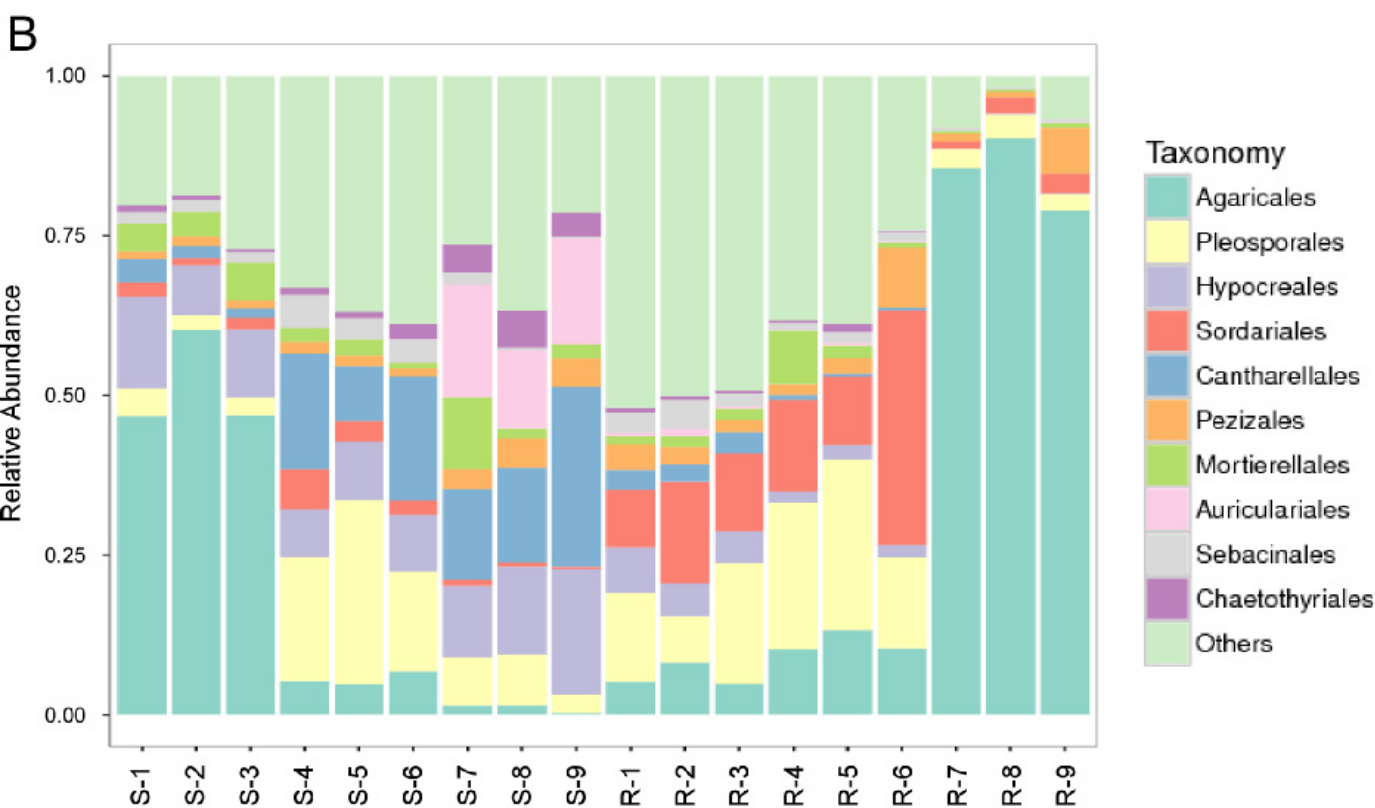

**Figure S4. Relative abundance of the ten most-abundant orders. (A) Bacteria. (B) Fungi.**

Supplement: Figure S4 — (A) Bacteria. (B)Fungi. [file peerj-07-8051-s004.pdf]

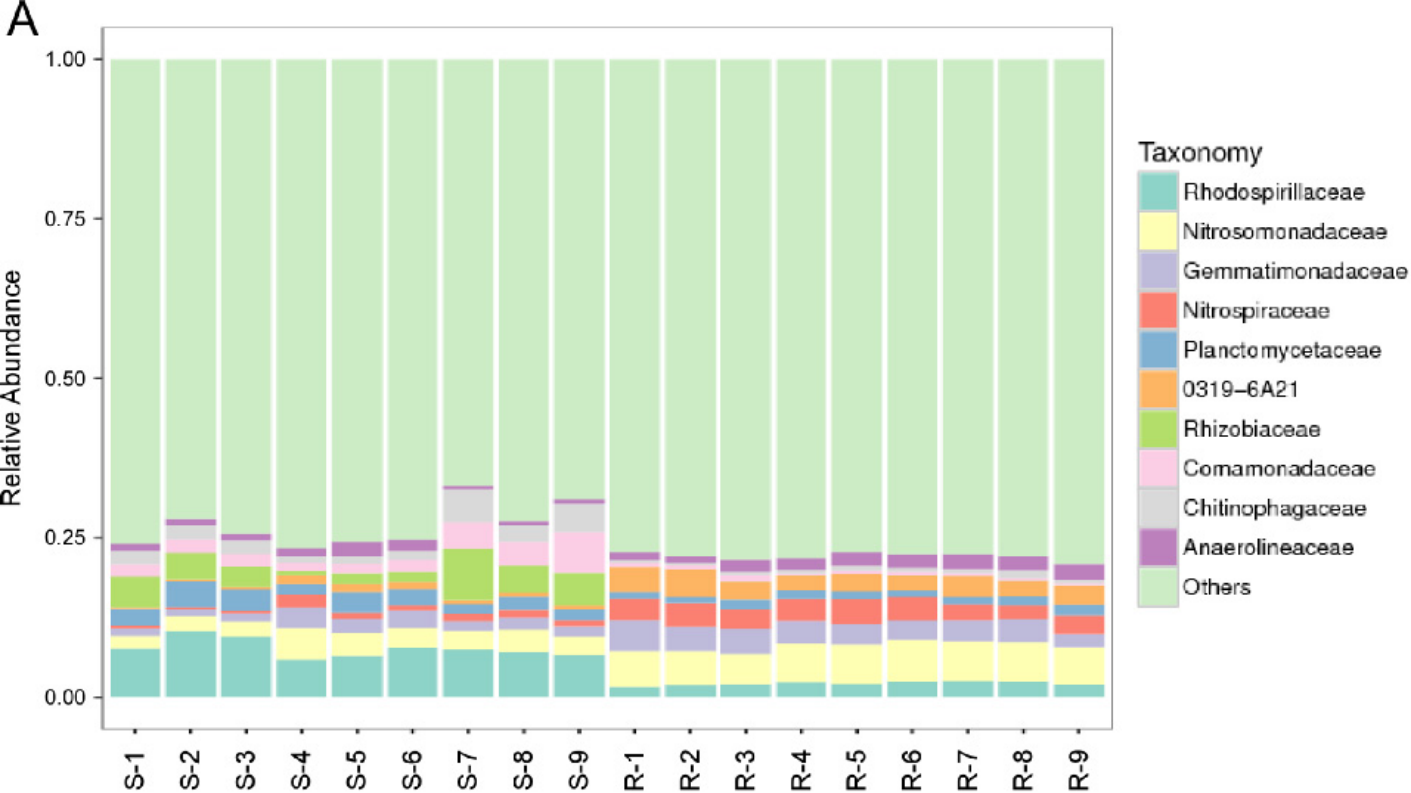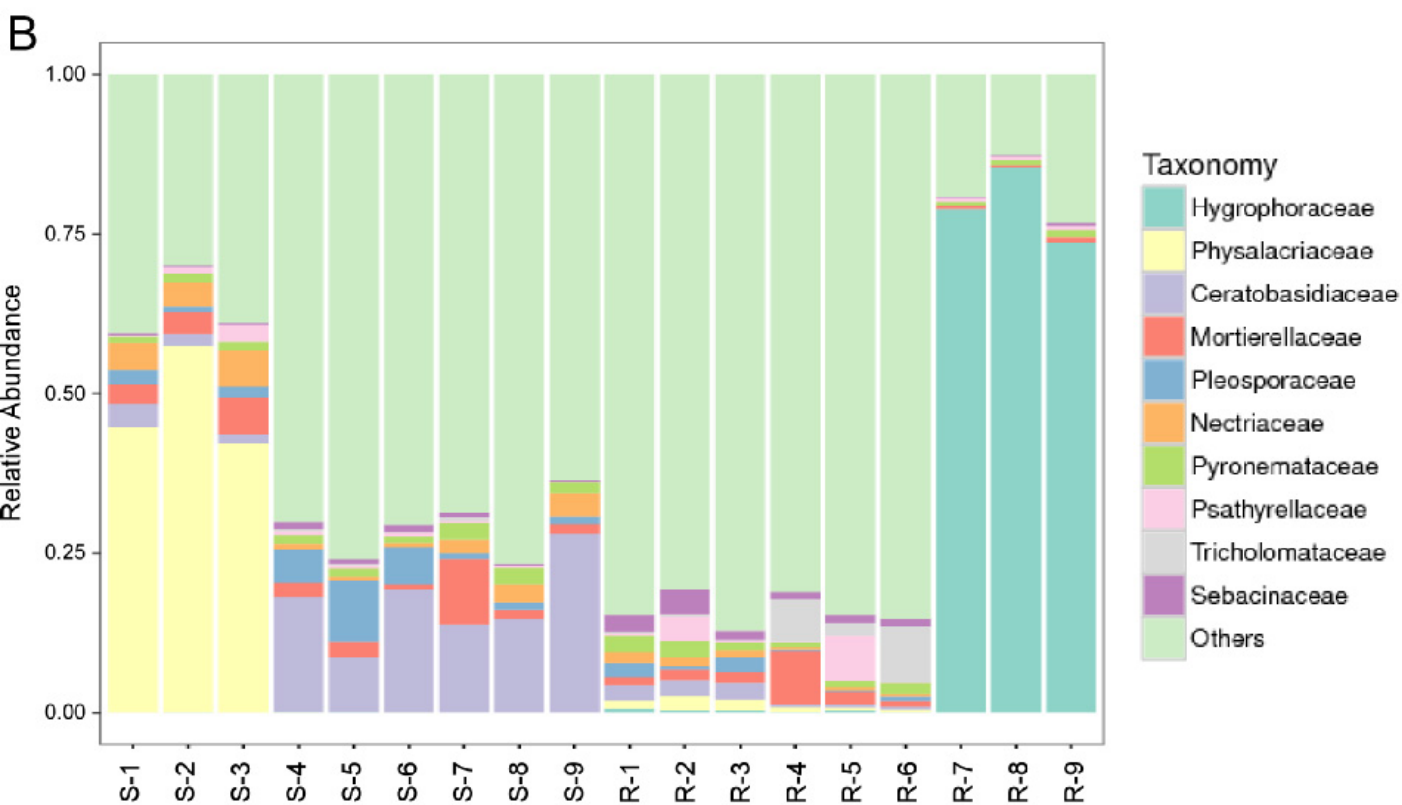

**Figure S5. Relative abundance of the ten most-abundant families. (A) Bacteria. (B) Fungi.**

Supplement: Figure S5 — (A) Bacteria. (B)Fungi. [file peerj-07-8051-s005.pdf]

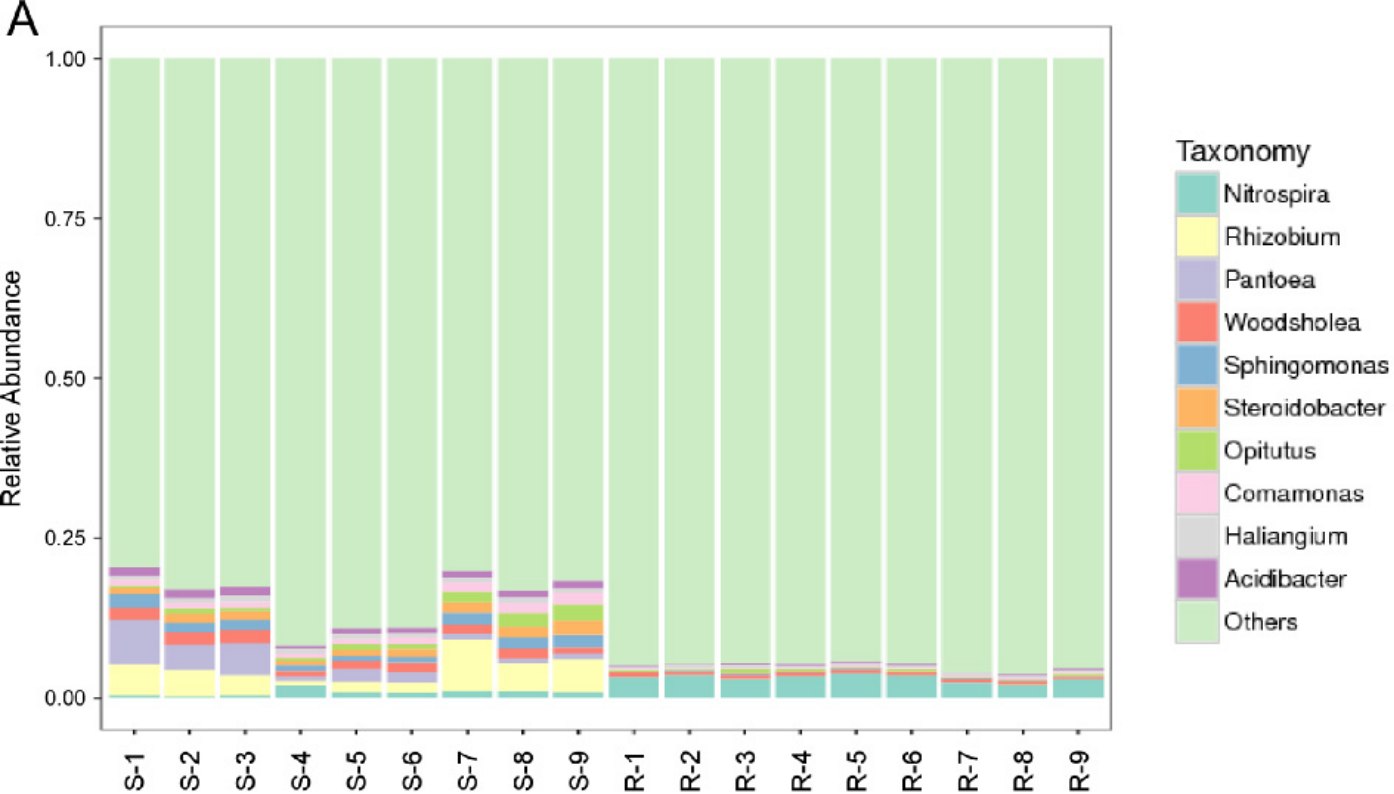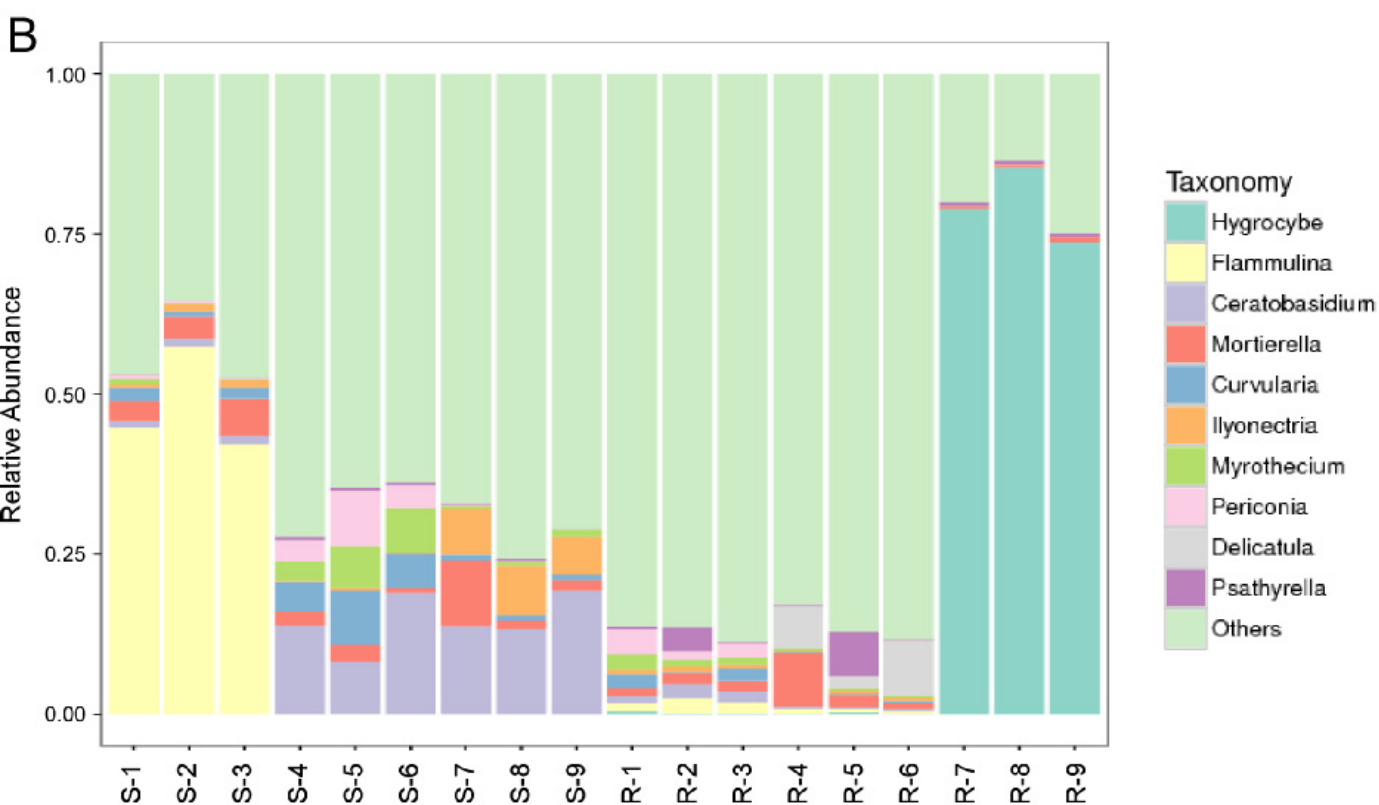

**Figure S6. Relative abundance of the ten most-abundant genera. (A) Bacteria. (B) Fungi.**

Supplement: Figure S6 — (A) Bacteria. (B)Fungi. [file peerj-07-8051-s006.pdf]
