# Supplementary material for: High-throughput sequence analysis reveals variation in the relative abundance of components of the bacterial and fungal microbiota in the rhizosphere of Ginkgo biloba
Source: PeerJ. 2019 Nov 15;7:e8051. doi: 10.7717/peerj.8051 (PMC6859886; doi:10.7717/peerj.8051)
Supplement: Figure S7 — (A) PCA analysis. Samples from the rhizosphere and the bulk soil are marked. (B) UPGMA cluster analysis. Samples collected from the rhizosphere are marked in blue, and samples collected from the bulk soil are marked in red. [file peerj-07-8051-s007.pdf]

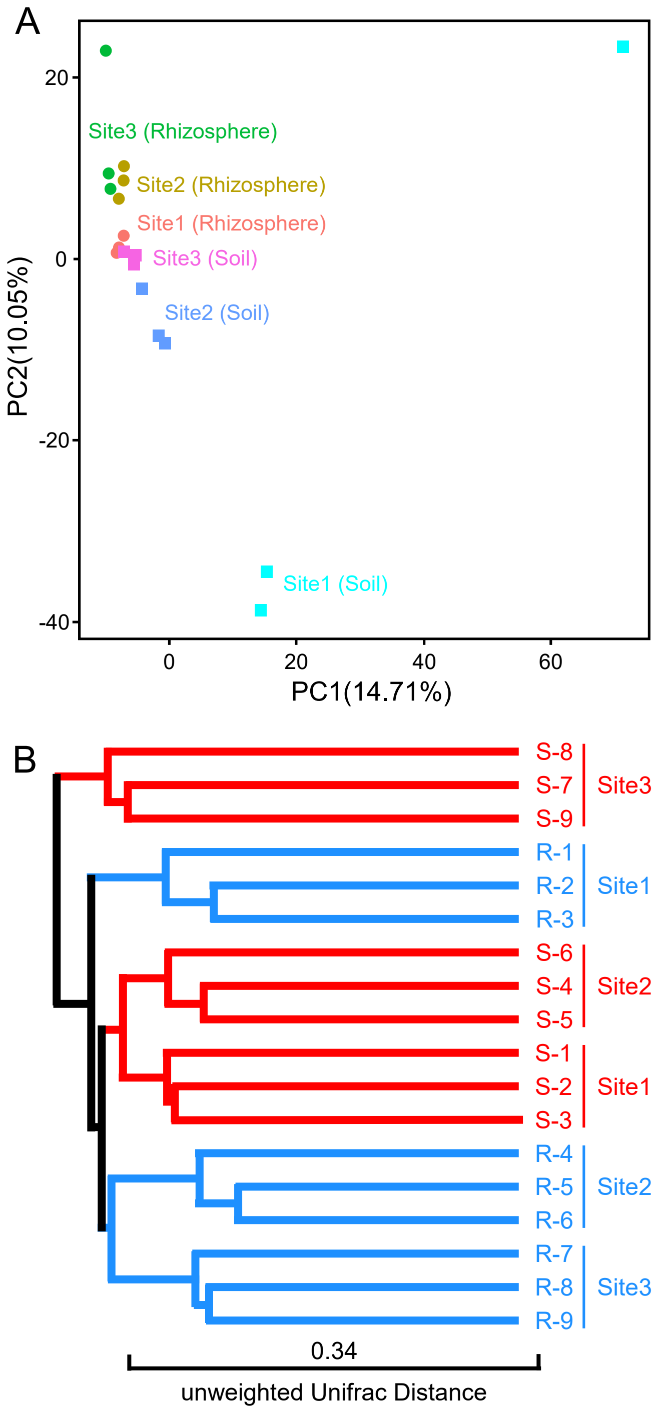

Figure S7. Beta diversity of the fungal communities. (A) PCA analysis. Samples from the rhizosphere and the bulk soil are marked. (B) UPGMA cluster analysis. Samples collected from the rhizosphere are marked in blue, and samples collected from the bulk soil are marked in red.
