# Supplementary material for: High-throughput sequence analysis reveals variation in the relative abundance of components of the bacterial and fungal microbiota in the rhizosphere of Ginkgo biloba
Source: PeerJ. 2019 Nov 15;7:e8051. doi: 10.7717/peerj.8051 (PMC6859886; doi:10.7717/peerj.8051)
Supplement: Figure S8 — The taxonomies of genera to the level of phylum are marked by different colors. The relative abundances of the 35 genera are scaled by a Z-score color gradient bar. The red colored data represent genera that have higher abundance than average. The blue colored data represent genera that have lower abundance than average. A Z-score of 0 represents a genus abundance value that is equal to the average abundance value. [file peerj-07-8051-s008.pdf]

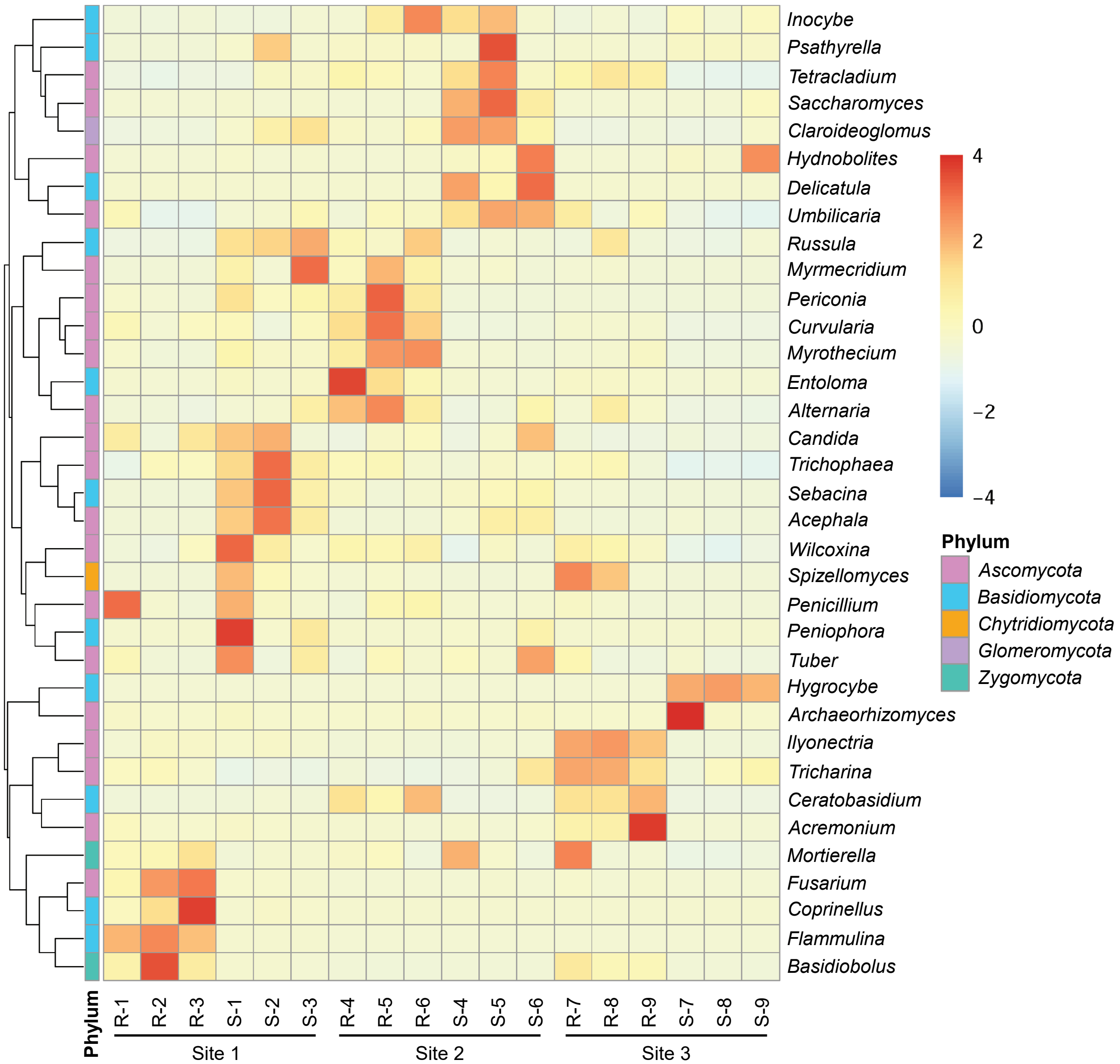

Figure S8. The abundance of the 35 most-abundant genera of fungi in the rhizosphere and the bulk soil from the three sites. The taxonomies of genera to the level of phylum are marked by different colors. The relative abundances of the 35 genera are scaled by a Z-score color gradient bar. The red colored data represent genera that have higher abundance than average. The blue colored data represent genera that have lower abundance than average. A Z-score of 0 represents a genus abundance value that is equal to the average abundance value.
