# Supplementary material for: High-throughput sequence analysis reveals variation in the relative abundance of components of the bacterial and fungal microbiota in the rhizosphere of Ginkgo biloba
Source: PeerJ. 2019 Nov 15;7:e8051. doi: 10.7717/peerj.8051 (PMC6859886; doi:10.7717/peerj.8051)
Supplement: Figure S9 — Each point represents a genus. The size and color of the spots represent the abundance of the genera. The relationships among correlated genera are represented by the size and color of lines. [file peerj-07-8051-s009.pdf]

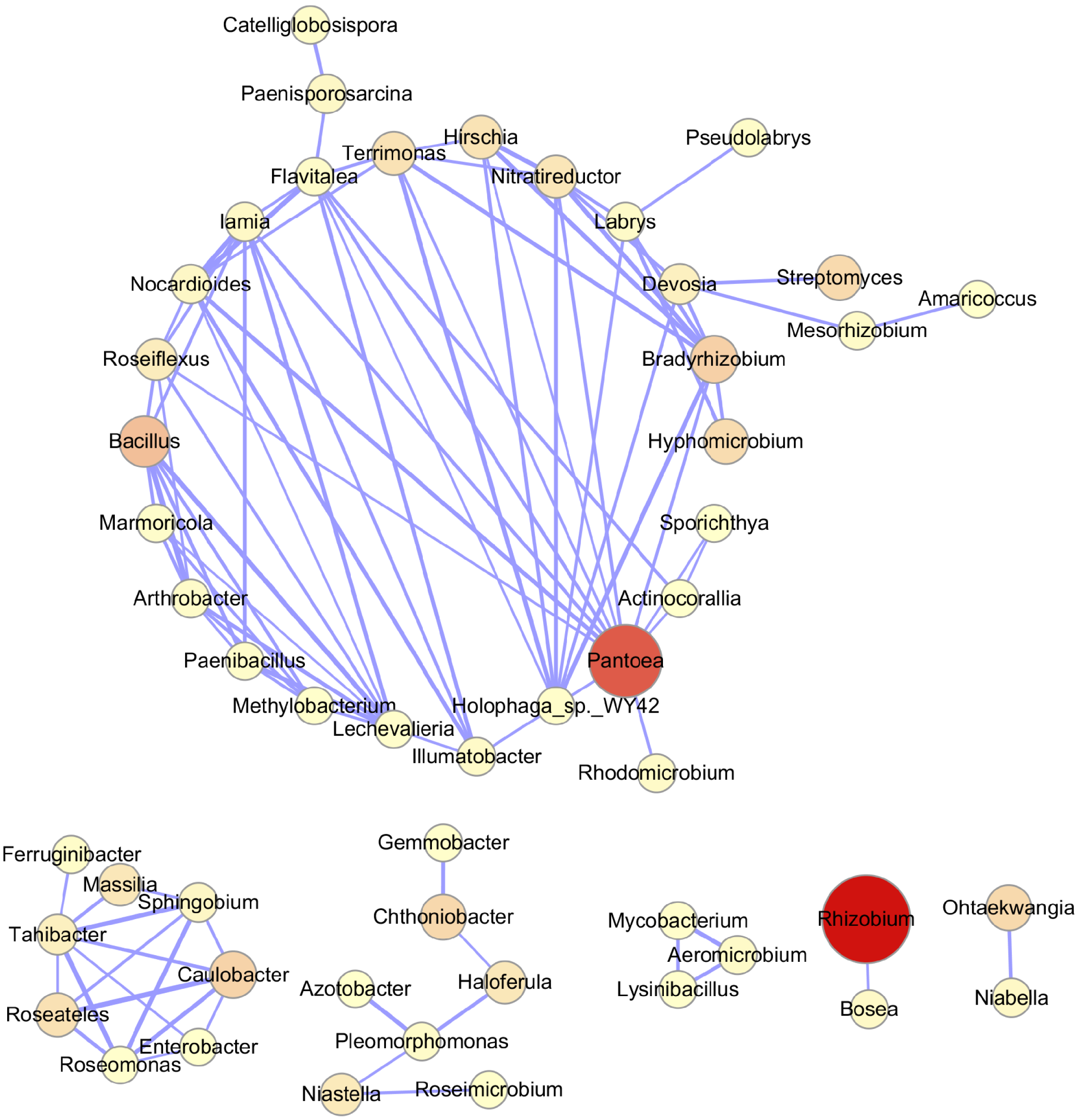

Figure S9. Correlation analysis in bacteria. Each point represents a genus. The size and color of the spots represent the abundance of the genera. The relationships among correlated genera are represented by the size and color of lines.
