# Supplementary material for: High-throughput sequence analysis reveals variation in the relative abundance of components of the bacterial and fungal microbiota in the rhizosphere of Ginkgo biloba
Source: PeerJ. 2019 Nov 15;7:e8051. doi: 10.7717/peerj.8051 (PMC6859886; doi:10.7717/peerj.8051)
Supplement: Figure S10 — Bars in the left, mean values of KEGG enrichment. Bars across circles, 95% confidence interval of the differences. Center of circles, differences of the mean values. Numbers in the right, p values of the significance test. [file peerj-07-8051-s010.pdf]

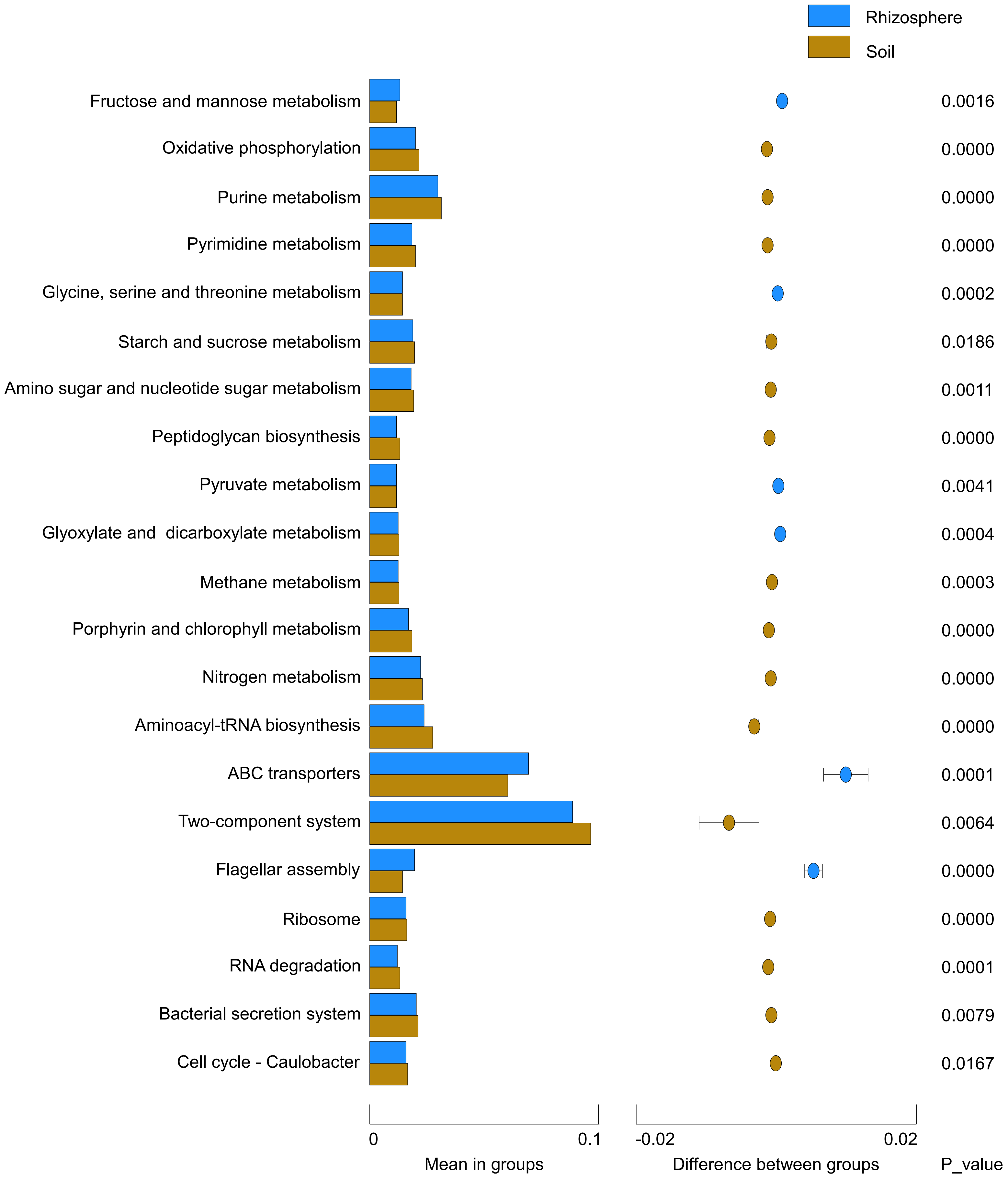


**Figure S10. KEGG functional analysis in bacteria.** Bars in the left, mean values of KEGG enrichment. Bars across circles, 95% confidence interval of the differences. Center of circles, differences of the mean values. Numbers in the right, p values of the significance test.
