# Supplementary material for: High-throughput sequence analysis reveals variation in the relative abundance of components of the bacterial and fungal microbiota in the rhizosphere of Ginkgo biloba
Source: PeerJ. 2019 Nov 15;7:e8051. doi: 10.7717/peerj.8051 (PMC6859886; doi:10.7717/peerj.8051)
Supplement: Table S1 [file peerj-07-8051-s011.pdf]

Provinces	Liaoning	Shandong	Jiangsu	Anhui	Hubei	Sichuan	Zhejiang	Guizhou
Cities	Dandong<br>Jinzhou	Rizhao<br>Yantai<br>Linyi	Taizhou<br>Yangzhou<br>Lianyungang<br>Yancheng<br>Rugao<br>Dongtai<br>Danyang<br>Taixing<br>Xinyi<br>Xuzhou	Huaibei<br>Suzhou<br>Guangming<br>Tianchang	Ezhou<br>Suizhou<br>Jingzhou<br>Anlu	Chengdu<br>Meishan<br>Guang'an<br>Shifang<br>Dazhou	Huzhou<br>Pinghu	Liupanshui<br>Bijie<br>Xingyi

Table S1. The name of cities treating *Ginkgo biloba* as a municipal tree.
