# Supplementary material for: High-throughput sequence analysis reveals variation in the relative abundance of components of the bacterial and fungal microbiota in the rhizosphere of Ginkgo biloba
Source: PeerJ. 2019 Nov 15;7:e8051. doi: 10.7717/peerj.8051 (PMC6859886; doi:10.7717/peerj.8051)
Supplement: Table S2 [file peerj-07-8051-s012.pdf]

| | Samples | Kingdom | Phylum | Class | Order | Family | Genus | Species |
|---|---|---|---|---|---|---|---|---|
| Site 1 | R-1 | 30857 | 30857 | 29978 | 29081 | 23944 | 16232 | 4653 |
| | R-2 | 25898 | 25898 | 25349 | 24421 | 20521 | 12426 | 3091 |
| | R-3 | 30274 | 30274 | 29639 | 28395 | 23124 | 13654 | 2978 |
| | S-1 | 25265 | 25265 | 24077 | 21058 | 12382 | 3898 | 239 |
| | S-2 | 22183 | 22183 | 21042 | 18458 | 10885 | 3550 | 199 |
| | S-3 | 29709 | 29709 | 28362 | 24868 | 14170 | 5044 | 447 |
| Site 2 | R-4 | 22398 | 22398 | 21573 | 19122 | 12833 | 5402 | 948 |
| | R-5 | 25620 | 25620 | 24645 | 22298 | 16371 | 7068 | 1086 |
| | R-6 | 28191 | 28191 | 27240 | 25097 | 18642 | 8081 | 1426 |
| | S-4 | 30053 | 30053 | 28275 | 24129 | 15097 | 4729 | 242 |
| | S-5 | 26394 | 26394 | 24756 | 21123 | 13654 | 4439 | 267 |
| | S-6 | 25629 | 25629 | 24055 | 20865 | 13619 | 4187 | 319 |
| Site 3 | R-7 | 24797 | 24797 | 24084 | 22663 | 18783 | 11754 | 3662 |
| | R-8 | 25363 | 25363 | 24657 | 22807 | 18159 | 10702 | 2488 |
| | R-9 | 24903 | 24903 | 24291 | 22785 | 19046 | 12319 | 4520 |
| | S-7 | 30256 | 30256 | 28443 | 24738 | 16000 | 4293 | 401 |
| | S-8 | 27511 | 27511 | 26159 | 22738 | 14283 | 4112 | 447 |
| | S-9 | 27550 | 27550 | 25904 | 21593 | 13470 | 4249 | 142 |

Table S2. Numbers of bacterial taxon tags at different levels of taxonomy.
