# Supplementary material for: High-throughput sequence analysis reveals variation in the relative abundance of components of the bacterial and fungal microbiota in the rhizosphere of Ginkgo biloba
Source: PeerJ. 2019 Nov 15;7:e8051. doi: 10.7717/peerj.8051 (PMC6859886; doi:10.7717/peerj.8051)
Supplement: Table S3 [file peerj-07-8051-s013.pdf]

	Samples	Kingdom	Phylum	Class	Order	Family	Genus	Species
Site 1	R-1	41897	40401	39539	37797	30933	27362	5463
	R-2	37683	35942	35118	34214	30554	29288	5495
	R-3	38279	36306	35428	32812	27955	26621	6879
	S-1	38319	36292	30563	28619	21642	18151	9691
	S-2	36522	33516	29613	24592	15849	14052	7957
	S-3	33911	32626	24229	22916	13103	10709	5778
Site 2	R-4	37702	35805	32986	27951	18856	15596	7629
	R-5	37499	34309	30289	27138	20991	19370	10891
	R-6	40269	36999	34407	28964	24039	21605	9326
	S-4	35793	34469	27680	25325	11568	10712	7734
	S-5	34558	33812	27537	25820	12279	11546	7473
	S-6	40297	39864	35345	34964	14595	13913	8003
Site 3	R-7	40769	37979	35171	34375	22547	19738	12087
	R-8	38240	34762	31360	27208	17663	14603	7882
	R-9	36227	34606	32155	30372	20051	14675	6517
	S-7	40608	40027	39614	39593	35880	35755	1739
	S-8	35124	34786	34677	34668	31066	31008	544
	S-9	41795	40417	39690	39623	35660	35412	1140

Table S3. Numbers of fungal taxon tags at different levels of taxonomy.
