# Supplementary material for: High-throughput sequence analysis reveals variation in the relative abundance of components of the bacterial and fungal microbiota in the rhizosphere of Ginkgo biloba
Source: PeerJ. 2019 Nov 15;7:e8051. doi: 10.7717/peerj.8051 (PMC6859886; doi:10.7717/peerj.8051)
Supplement: Table S4 [file peerj-07-8051-s014.pdf]

	Samples	PD_whole_tree	Observed_species	Shannon	Simpson	ACE	Chao1	Goods_coverage
Site 1	R-1	104	2076	8.364	0.985	2972	2887	0.966
	R-2	104	2103	8.615	0.987	2980	3054	0.966
	R-3	110	2186	8.706	0.988	3059	3051	0.965
	S-1	139	2865	9.584	0.995	3926	3862	0.956
	S-2	130	2678	9.471	0.995	3624	3551	0.96
	S-3	141	2934	9.571	0.994	3960	3935	0.955
Site 2	R-4	133	2799	9.671	0.995	3756	3711	0.959
	R-5	135	2861	9.721	0.995	3851	3848	0.957
	R-6	135	2776	9.565	0.994	3842	3763	0.957
	S-4	146	2890	9.708	0.996	4029	4003	0.954
	S-5	143	2841	9.676	0.996	3970	3848	0.955
	S-6	149	2822	9.698	0.996	3794	3687	0.958
Site 3	R-7	127	2605	9.06	0.992	3827	3716	0.955
	R-8	129	2676	9.39	0.995	3710	3677	0.958
	R-9	123	2527	9.033	0.992	3577	3462	0.959
	S-7	133	2528	9.375	0.995	3472	3393	0.961
	S-8	142	2779	9.686	0.996	3832	3838	0.957
	S-9	144	2776	9.615	0.996	3848	3775	0.956

Table S4. Alpha diversity analysis of the bacterial communities.
