# Supplementary material for: High-throughput sequence analysis reveals variation in the relative abundance of components of the bacterial and fungal microbiota in the rhizosphere of Ginkgo biloba
Source: PeerJ. 2019 Nov 15;7:e8051. doi: 10.7717/peerj.8051 (PMC6859886; doi:10.7717/peerj.8051)
Supplement: Table S5 [file peerj-07-8051-s015.pdf]

	Samples	PD_whole_tree	Observed_species	Shannon	Simpson	ACE	Chao1	Goods_coverage
Site 1	R-1	252	434	4.38	0.789	476	471	0.998
	R-2	198	333	3.574	0.666	366	370	0.998
	R-3	197	322	4.341	0.811	346	358	0.999
	S-1	384	731	7.216	0.975	744	747	0.999
	S-2	309	529	6.345	0.959	539	547	0.999
	S-3	317	564	5.955	0.928	583	599	0.999
Site 2	R-4	269	465	5.928	0.961	505	517	0.998
	R-5	306	545	5.992	0.964	580	585	0.998
	R-6	295	527	5.738	0.944	557	561	0.998
	S-4	214	337	4.801	0.922	348	345	0.999
	S-5	254	426	5.195	0.935	451	463	0.999
	S-6	281	455	4.449	0.841	467	471	0.999
Site 3	R-7	305	545	5.655	0.942	586	588	0.998
	R-8	265	460	5.505	0.949	486	483	0.998
	R-9	240	412	5.004	0.93	454	464	0.998
	S-7	144	235	1.691	0.373	272	265	0.998
	S-8	101	143	1.131	0.267	192	188	0.999
	S-9	129	185	1.904	0.45	229	234	0.998

Table S5. Alpha diversity analysis of the fungal communities.
